# Mitochondrial Dynamics and SLC25 Transporters in Neurodegeneration: From Mechanisms to Therapeutic Opportunities

**DOI:** 10.3390/biom16060842

**Published:** 2026-06-09

**Authors:** Giampaolo Morciano, Ruggiero Gorgoglione, Vito Porcelli, Amer Ahmed, Pasquale Scarcia, Angelo Vozza, Francesco Massimo Lasorsa, Giuseppe Fiermonte, Luigi Palmieri

**Affiliations:** 1 Department of Biosciences, Biotechnologies and Environment, University of Bari Aldo Moro, 70125 Bari, Italy; vito.porcelli@uniba.it (V.P.); amer.ahmed@uniba.it (A.A.); pasquale.scarcia@uniba.it (P.S.); angelo.vozza@uniba.it (A.V.); francesco.lasorsa@uniba.it (F.M.L.); giuseppe.fiermonte@uniba.it (G.F.); 2 Department of Medicine and Surgery, LUM University, 70010 Casamassima, Italy; gorgoglione@lum.it

**Keywords:** neurodegeneration, mitochondrial dynamics, SLC25 carriers, metabolism

## Abstract

Neurodegenerative diseases are increasingly recognized as disorders of due to disrupted cellular homeostasis, with mitochondrial dysfunction playing a central and early role in disease progression. This review explores the intricate relationship between mitochondrial function and neuronal health, emphasizing the pivotal role of the solute carrier family 25 (SLC25) transporters in maintaining mitochondrial homeostasis. We provide a comprehensive overview of mitochondrial biology in the central nervous system, including energy metabolism, calcium signaling, redox regulation, organelle interactions and mitochondrial dynamics. We delve into the SLC25 transporter family, highlighting their transport mechanisms, substrates and roles in brain metabolism and neuroprotection. SLC25 on one hand and proteins involved in the regulation of mitochondrial morphology and calcium signaling on the other hand are two sides of the same coin influencing each other. A critical analysis follows, examining how mitochondrial dysfunction contributes to mitochondrial abnormalities in a spectrum of neurodegenerative diseases, including Alzheimer’s disease, Parkinson’s disease, ALS and rare mitochondrial encephalopathies. Finally, we assess emerging therapeutic strategies targeting mitochondrial pathways and SLC25 function, including metabolic modulation, gene therapies, antioxidants and pharmacological agents. This review underscores mitochondria and the SLC25 transporters as promising targets for disease-modifying interventions in neurodegeneration and raises key questions about the causality between mitochondrial failure and neuronal death.

## 1. Introduction

Neurodegenerative diseases (NDs) comprise a broad and heterogeneous group of disorders characterized by progressive loss of neuronal structure and/or function within specific regions of the nervous system, ultimately culminating in cell death [1,2].

The progressive nature of neurodegeneration leads to cognitive decline, motor impairments and significant loss of autonomy and quality of life for affected individuals. The increasing prevalence of NDs, largely driven by global aging demographics, presents significant challenges to healthcare infrastructure and socioeconomic systems [3]. This trend underscores the urgent need to unravel the complex molecular and cellular mechanisms that drive neurodegeneration.

NDs encompass common conditions such as Alzheimer’s disease (AD), Parkinson’s disease (PD) and Amyotrophic Lateral Sclerosis (ALS), as well as less prevalent disorders including Huntington’s disease (HD) and rare mitochondrial encephalopathies [2]. Despite differences in prevalence, clinical manifestations and affected brain regions, NDs share common pathological features including neuroinflammation, protein misfolding, oxidative stress and mitochondrial dysfunction.

Mitochondria participate in a wide range of metabolic and signaling processes. They have a central role in cellular bioenergetics, as several key pathways, including the tricarboxylic acid (TCA) cycle, β-oxidation of fatty acids (FA) and amino acid transamination, occur almost exclusively within the mitochondrial matrix. Beyond their metabolic functions, mitochondria are critical for intracellular calcium (Ca^2+^) buffering, thereby ensuring Ca^2+^ signaling that underlies cellular excitability and communication [4,5]. They also act as gatekeepers of apoptosis by regulating the release of cytochrome c and other pro-apoptotic factors, effectively linking the energetic state of the cell to programmed cell death [4,6]. Moreover, mitochondria maintain intimate cross-talk with other organelles, particularly the endoplasmic reticulum (ER), peroxisomes and lysosomes, coordinating processes such as lipid metabolism, Ca^2+^ exchange, autophagy and stress responses [7,8]. Importantly, mitochondrial function is regulated by several mitochondrial quality control mechanisms (mQCM) which finely tune their fitness [9]. All these features underscore mitochondria as dynamic signaling hubs rather than mere energy producers [1].

On their parts, neurons are uniquely vulnerable because of their post-mitotic nature, polarized morphology and extensive synaptic networks that impose exceptionally high energetic demands. To maintain excitability and plasticity, mitochondria generate ATP through oxidative phosphorylation (OXPHOS), which supports ion pumping, neurotransmitter release and axonal function. Beyond energy metabolism, neuronal mitochondria buffer Ca^2+^ transients during synaptic activity and regulate intrinsic apoptotic pathways. Even subtle impairments in these processes can compromise neurotransmission, increase oxidative stress and predispose neurons to degeneration [1]. Other brain cell populations (i.e., astrocytes, oligodendrocytes) are equally dependent on intact mitochondrial function, although their contributions differ from those of neurons.

The finely tuned mitochondrial functions depend heavily on the selective permeability of the inner mitochondrial membrane (IMM), which is primarily, but not exclusively, mediated by a large family of membrane transport proteins known as the solute carrier family 25 (SLC25) [10]. In humans, this family comprises 53 proteins, many of which have been functionally characterized as transporters of nucleotides, cofactors, carboxylates, amino acids and inorganic ions [11,12]. Given the large number of proteins in the SLC25 family, the wide range of metabolic and signaling pathways in which they participate, and the centrality of mitochondria to cellular physiology, it is unsurprising that dysfunction of these transporters is associated with NDs. In particular, members involved in ATP/ADP exchange (adenine nucleotide transporters, ANTs) [13,14], iron homeostasis (mitoferrins) [15], as well as mitochondrial carrier homolog (MTCH) 1 and 2 [16] have been consistently implicated across AD, PD, ALS and HD.

This review provides a comprehensive assessment of mitochondrial dysfunction in NDs, with a particular focus on the contribution of members of the SLC25 family and their possible relation with mQCM. We begin by outlining structural and mechanistic features of the SLC25 family with multiple references to cellular metabolism, followed by the importance of mitochondrial physiology in the brain. Subsequently, we examine genetic and pathological evidence linking SLC25 and mQCM to neurodegenerative phenotypes. Finally, we explore emerging therapeutic strategies targeting SLC25- and mQCM-mediated pathways and their potential to preserve mitochondrial function and mitigate neurodegeneration. In contrast, the literature on NDs associated with mitochondrial DNA mutations is limited here, as both the conditions and the related therapeutic interventions have been extensively reviewed elsewhere [17,18,19,20,21,22].

## 2. SLC25 Carrier Family

### 2.1. Overview of the SLC25 Family and Their Transport Mechanisms

SLC25, also known as Mitochondrial Carriers Family (MCF), represents the largest and most functionally diverse group of metabolite transporters in the IMM. Notably, the highly selective permeability of IMM is not mediated solely by SLC25 carriers; other transport families with distinct structural and functional properties also participate [10].

SLC25 members share the same characteristic tripartite architecture composed of three tandemly repeated homologous domains of approximately 100 amino acids each [23].

Within each repeat, a consensus sequence motif P-X-[D/E]-X-X-[K/R]-X-[R/K] (20–30 residues) [D/E]-G-X-X-X-X-[W/Y/F]-[K/R]-G is typically present, though not always fully conserved [24]. Each domain consists of an odd-numbered transmembrane α-helix, followed by a loop containing a short matrix α-helix oriented parallel to the membrane plane, and an even-numbered transmembrane α-helix. These three domains are arranged to form a barrel-shaped structure composed of six transmembrane α-helices, exhibiting threefold pseudo-symmetry.

The substrate-binding site of SLC25 carriers is located at the midpoint of the central cavity and alternately faces the intermembrane space or the matrix, consistent with the alternating-access mechanism. The binding site comprises three contact points (CPs) on the even-numbered helices: CP II defines substrate class (e.g., keto acids versus amino acids), CP I refines specificity within a class and CP III is a conserved positive charge [25]. At both ends of the carrier, salt-bridge networks act as molecular gates, switching between the cytoplasm-open state (c-state) and matrix-open state (m-state). They operate predominantly as strict counter-exchangers (antiporters) of structurally related substrates, though some function as unidirectional transporters (uniporters) or mediate substrate (symport).

### 2.2. Key Substrates Transported

The human SLC25 family comprises 53 members that transport a wide range of metabolites, including nucleotides, amino acids, FA, cofactors and inorganic ions (Table 1). Here, we provide an overview of main substrates and functions of those carriers that are mentioned in this review, while additional descriptions can be found in specialized reports by our group [11,12,26] and others [27,28].

The ADP/ATP carriers (ANT1–4; SLC25A4, SLC25A5, SLC25A6, SLC25A31, respectively) sustain cellular energy homeostasis by exchanging mitochondrial ATP, the energy currency of the cell, for cytosolic ADP [29,30]. The ATP-Mg/Pi carriers (APC1–4; SLC25A24, SLC25A23, SLC25A25, SLC25A41, respectively) mediate electroneutral phosphate–adenine nucleotide magnesium exchange [31]. AGC1/2 (SLC25A12, SLC25A13) catalyze Ca^2+^-regulated aspartate–glutamate exchange within the malate–aspartate shuttle (MAS) [32,33,34], while GC1/2 (SLC25A22, SLC25A18) mediate glutamate import with a proton [35]. GLYC (SLC25A38) provides glycine for heme biosynthesis [36] and ORNT1/2 (SLC25A15, SLC25A2) exchange ornithine and citrulline in the urea cycle [37,38]. Also, SLC25A29 is abundantly expressed in the human brain, with particularly high levels in the cerebral cortex, cerebellum, hippocampus and hypothalamus. It primarily imports basic amino acids, such as arginine and lysine, into mitochondria to support cellular metabolism and protein synthesis [39]. One of the most recently identified is SLC25A44, described as a branched-chain amino acid transporter despite some disputes currently being discussed [40]. In addition, several carriers import vitamins and cofactors required for mitochondrial metabolism, such as the thiamine pyrophosphate carrier (TPC; SLC25A19) [41], the flavin/folate carrier (SLC25A32) [42] and the coenzyme A transporter (SLC25A42) [43]. Inorganic ion transport is of crucial importance and is mediated by the mitoferrins (SLC25A37, SLC25A28), which deliver iron for heme and Fe–S cluster synthesis [44], and by the phosphate carrier PiC (SLC25A3), which provides phosphate for ATP production [45].

The carnitine/acylcarnitine carrier CAC (SLC25A20) is essential for FA β-oxidation by exchanging acyl-carnitine for carnitine [46]. Additional metabolite fluxes are mediated by the dicarboxylate carrier DIC (SLC25A10), which transports malate, succinate and related compounds [47]; the oxoglutarate carrier OGC (SLC25A11) [48] and the oxodicarboxylate carrier ODC (SLC25A21), which exchange oxoacids critical for shuttles and amino acid catabolism [49]; and the citrate carrier CIC (SLC25A1), which exports citrate or isocitrate in exchange for other tricarboxylates, dicarboxylates or phosphoenolpyruvate, thereby linking mitochondrial and lipid metabolism [50].

The uncoupling proteins (UCP1–6; SLC25A7, SLC25A8, SLC25A9, SLC25A27, SLC25A14, SLC25A30) form a distinct subfamily closely related to UCP1 which is responsible for adaptive thermogenesis in brown adipose tissue (BAT) by dissipating the proton gradient. Biochemical evidence suggests that they predominantly act as carboxylate transporters, consistent with their phylogenetic proximity to dicarboxylate carriers [51,52].

SLC25A51 (MCART1) encodes a mitochondrial carrier protein whose main function is to transport NAD^+^ from the cytosol into the mitochondrial matrix [53]. NAD^+^ is an essential cofactor required for several metabolic reactions involved in cellular energy production. Since mitochondria cannot synthesize sufficient amounts of NAD^+^ on their own and the molecule cannot freely cross the inner mitochondrial membrane, SLC25A51 plays a critical role in maintaining the mitochondrial NAD^+^ pool. More recently, it has attracted attention because mitochondrial NAD^+^ metabolism is closely linked to aging, neurodegeneration and other metabolic disorders.

Finally, a subset of SLC25 proteins lack a defined transport activity but play roles in mitochondrial dynamics and apoptosis. MTCH1/2 (SLC25A49, SLC25A50) are outer membrane proteins acting as receptor-like components of apoptotic signaling [54], while SLC25A46 regulates mitochondrial network organization [55]. Several additional members, including SLC25A34, SLC25A45 and SLC25A53, remain functionally orphaned, while SLC25A35 was characterized a few months ago and regulates mitochondrial phosphoenolpyruvate efflux and glyceroneogenesis in lipogenic cells that utilize the pyruvate-to-PEP bypass [56].

## 3. Mitochondrial Function in the Brain: A Crosstalk Between Metabolism and Mitochondrial Morphology

The importance of mitochondria in maintaining brain function lies in their morphology, featured by two phospholipidic membranes, which delimit two areas named as the intermembrane space (IMS) and the matrix. The matrix serves as the primary site for mitochondrial activity, housing numerous enzymes essential for metabolism and cell life; this is assisted by the selective permeable IMM which encircle the matrix and provide for a high number of specific carriers for small molecules and ions, besides the complexes of the electron transport chain (ETC).

Mitochondria are the primary sites of ATP production, supplying the energy required for synaptic transmission [57], ion gradient maintenance [58] and neurotransmitter synthesis [59]. In the brain, glucose metabolism through glycolysis provides pyruvate, which enters mitochondria and fuels the TCA cycle. This catabolic process is almost exclusively the unique source of feeding and generates reducing equivalents (NADH, FADH_2_) that drive the ETC culminating in ATP synthesis.

However, the brain is composed of several cell types’ populations with a significant degree of metabolic diversity. Neurons rely heavily on OXPHOS to meet their energy demands, even though somata own higher levels of aerobic glycolysis compared to axon terminals, as a mechanism to prevent oxidation [60]. Similarly, microglia primarily depend on OXPHOS for energy but can shift to a glycolysis-dominant metabolic state, driving certain neurological conditions [61]. Oligodendrocytes predominantly use glycolysis to generate ATP during development and remyelination; but they can export pyruvate or lactate for energy requirements to surrounding axons [62]. Also, astrocytes use glycolysis converting glucose into lactate with minimal oxygen use. This lactate is then transferred to neurons, where it is fully oxidized [63]. Microglial mitochondria, by contrast, act as metabolic regulators of immune activity, shaping responses to stress and injury through redox signaling, production of reactive oxygen species, and control of pro- or anti-inflammatory states [1].

**Figure 1:**
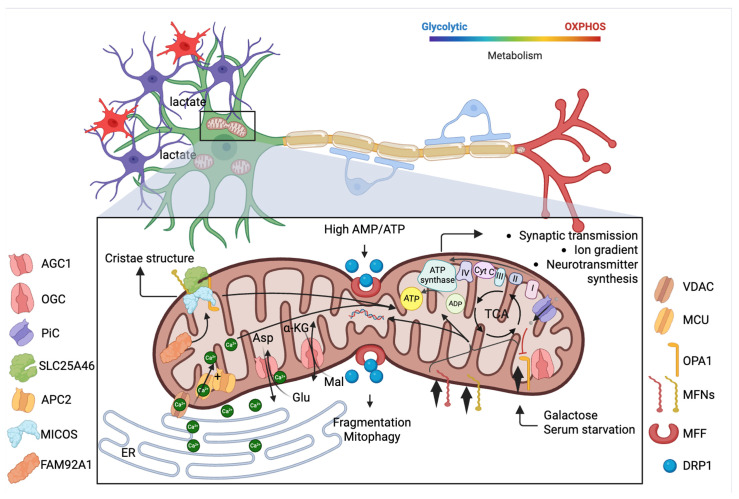
Schematic representation of mitochondrial structure, dynamics and metabolism in brain cells highlighting the role of key mitochondrial carriers and structural proteins. AGC1 (SLC25A12) participates in the Ca^2+^-regulated malate–aspartate shuttle supporting NADH transport and neuronal protection. OGC (SLC25A11) mediates α-ketoglutarate/malate exchange and interacts with OPA1 to regulate cristae remodeling during nutrient stress. PiC (SLC25A3) imports phosphate and copper, modulating redox-dependent mitochondrial “flickering” and OPA1 processing. SLC25A46 maintains mitochondrial lipid homeostasis and cristae architecture by interacting with MFN2, OPA1 and the MICOS complex. MCU governs Ca^2+^ uptake from mitochondria-associated membranes (MAMs), coupling neuronal activity to ATP synthesis. APC2 supports ATP/ADP exchange and enhances mitochondrial Ca^2+^ uptake via interaction with MCU and MICU1. The MICOS complex organizes cristae junctions, while FAM92A1 shapes inner membrane curvature and supports synaptic structure. Mitochondrial dynamics are driven by MFNs and OPA1 (fusion) and DRP1 (fission), ensuring proper morphology, bioenergetic efficiency and neuronal function. α-KG: α-ketoglutarate; Asp: aspartate; ER: endoplasmic reticulum; Glu: glutamate; Mal: malate. Red cells: microglia; Blue cells: astrocytes. Bolded black arrows: increased protein expression. Created with BioRender.com.

Moreover, brain metabolism changes with age with a maximum peak of glucose utilization during early development [64] and a global decrease in both lactate production and use of monocarboxylates in aging [65]; with a more pronounced reduction in glucose uptake compared to oxygen consumption (Figure 1). Importantly, mitochondrial metabolism is also involved in producing key biosynthetic precursors such as amino acids, lipids and nucleotides that are essential for neural development and plasticity. As a consequence, dysregulation of mitochondrial metabolism can impair synaptic function [66]. For instance, reduced ATP production compromises Na^+^/K^+^-ATPase activity, leading to altered neuronal excitability. Moreover, defects in mitochondrial metabolic enzymes have been implicated in various NDs, including Leigh syndrome and mitochondrial encephalomyopathies.

A few months ago, some researchers added complexity to this scenario. They described substantial variability in mitochondrial characteristics across different brain regions, through the establishment of a MitoBrainMap, by integrating mitochondrial measurements with brain imaging data (MRI and PET) from nearly 2000 living adults [1]. Notably, grey matter was found to contain over 50% more mitochondria than white matter, and the mitochondria in grey matter were more specialized for high energy production. Evolutionary differences also appeared: recently evolved cortical areas such as the frontal and temporal lobes exhibited mitochondria that were biochemically optimized for high-performance energy metabolism, reflecting the increased energy demands of complex cognitive functions in these regions [1].

Mitochondrial morphology is dynamic and adapts to different conditions; moreover, it shapes the function of the organelle [67]. Mitochondrial shape, size and distribution inside cells are the consequence of fusion and fission processes and are strongly linked to cell fate in neurogenesis [68]. It has been shown that cells destined to self-renew undergo mitochondrial fusion, whereas those that retain high levels of mitochondria fission become neurons [68]. Mitochondrial dynamics also significantly impact metabolism. Fusion allows mitochondria to mix their contents, including metabolites and mtDNA, mitigating damage and optimizing energy production [69,70]. Proteins such as mitofusins (MFN1/2) in the OMM and optic atrophy 1 (OPA1) in the IMM orchestrate this process. Their importance in brain physiology is documented by multiple studies in which dysfunctional and mutated isoforms expression are lethal during early life stages onset and can significantly prompt neurodegeneration after maturation [71,72], concomitantly to Complex I deficiency, reduced mitochondrial potential (ΔΨ_m_) and respiration, impaired ability to perform mitophagy during stress [73,74] and Ca^2+^ dyshomeostasis [74].

Conversely, fission enables mitochondrial division, facilitating the removal of damaged mitochondria through mitophagy [75,76] and allowing proper distribution along axons and dendrites. Dynamin-related protein 1 (DRP1) and mitochondrial fission factor (MFF) are essential in this mechanism. For instance, when cells experience energy stress, the AMP/ATP ratio increases, leading to activation of AMP-activated protein kinase (AMPK). This is both necessary and sufficient to trigger mitochondrial fragmentation through the phosphorylation of MFF, which is essential for the recruitment of DRP1 on the outer membrane of mouse cortical neurons. This mechanism is an example of connection between bioenergetics and mitochondrial morphology, which helps distribute energy locally with fission, and mitophagy for the selective removal of damaged mitochondria with fusion [77].

This molecular partnership directly entails the SLC25 transporters, although they remain poorly studied in this respect. In 2014, Patten D. and colleagues revealed how mitochondrial architecture dynamically adapts to shifts in metabolic conditions, with a central role played by OPA1 [78]. When cells are subjected to conditions of nutrient scarcity, such as growth in galactose (which forces reliance on OXPHOS) or complete starvation, OPA1 forms higher-order oligomers tightening the cristae junctions, leading to enhanced assembly of ATP synthase oligomers and respiratory chain supercomplexes. This remodeling process is intimately linked to the SLC25 family transporters, which are required for the oligomerization of OPA1. Indeed, knockdown of *SLC25A11*, whose encoded protein would physically interact with OPA1, prevented from forming the oligomers necessary for cristae tightening with defects in ATP production [78]. Otherwise, prolonged mitochondrial hyperfusion can be harmful for the cell. In this context, ten years after the first finding about SLC25 and mitochondrial dynamic proteins interactions, it has been described another type of cooperation between OPA1 and PiC [79]. One of the mechanisms controlling flickering, a phenomenon consisting of transient mitochondrial depolarization events as a signal to activate the protease Oma1-dependent OPA1 processing to suppress further fusion, involves PiC. PiC has been demonstrated to be involved in the import of both copper and phosphate into mitochondria [80,81]. The study demonstrated that PiC-mediated copper transport or copper deficiency leads to a failure of flickering. Indeed, copper is essential for the activity of enzymes such as cytochrome c oxidase (COX) and superoxide dismutase 1 (SOD1), both of which are crucial for maintaining redox balance. Thus, mitochondrial flickering acts as a redox-sensitive signal to trigger a protective response, ensuring that mitochondrial dynamics remain balanced under metabolic stress [79].

Similarly, while not a classical SLC25 family member in terms of substrate specificity, SLC25A46 plays a role in mitochondrial lipid homeostasis and cristae maintenance [82]. SLC25A46 is localized at the OMM and there, interacts with several key regulators of mitochondrial architecture and dynamics, including MFN2, OPA1 [83], the mitochondrial contact site and cristae organizing system (MICOS) complex [84] and the ER membrane complex (EMC) that controls mitochondrial organization [82].

Structural adjustments of mitochondria also involve cristae remodeling, which is closely linked to the organelle’s metabolic state and may include variations in cristae number and spacing between membranes. Researchers have demonstrated that the shape of the cristae directly influences the formation and stability of respiratory supercomplexes, facilitating electron transfer and proton pumping, thereby optimizing ATP production [85]. Vice versa, energy-demanding situations promote cristae formation [86]. The importance of this parameter in brain physiology was further reported recently through the investigation of a mitochondrial protein function, FAM92A1, which is expressed in neurons from embryonic stages and is crucial for maintaining mitochondrial morphology. The absence of FAM92A1 results in disrupted mitochondrial IMM structures, including abnormal cristae formation. These mitochondrial defects are associated with impaired synaptic function, as evidenced by changes in synaptic vesicle morphology and synaptic plasticity. Furthermore, *FAM92A1* knockout mice exhibit cognitive deficits and neuronal degeneration [87]. Indeed, FAM92A1 interacts with phosphoinositide- and cardiolipin-containing membranes, inducing lipid clustering and membrane curvature (Figure 1).

The dynamic structure of mitochondria allows engaging temporary physical and functional contact sites with other organelles, particularly with the ER, through structures known as mitochondria-associated membranes (MAMs). These contact sites are crucial for lipid transfer, Ca^2+^ signaling and mitochondrial division thus providing a further implication with bioenergetics [8]. But mitochondria also interface with plasma membranes through Rhotekin2, whose binding directs the organelle localization close to some specific dendritic sites, essential for dendritic branch induction [88].

In neurons, MAMs facilitate rapid Ca^2+^ exchange during action potential firing, ensuring precise regulation of neurotransmitter release and synaptic plasticity. Using in situ electrophysiology and two-photon imaging has been monitored mitochondrial calcium uniporter (MCU) activation to allow the entry of Ca^2+^ from MAMs into mitochondria, during high-frequency action potential firing. This activation enhanced ETC activity and promoted the reduction in NAD^+^ to NADH, thereby supporting ATP production [89]. Additionally, mitochondrial Ca^2+^ buffering attenuates cytosolic Ca^2+^ signals, reducing the coupling between neuronal activity and the slow afterhyperpolarization, a key regulator of neuronal excitability. The study also highlights regional differences in the strength of the relationship between firing rate and mitochondrial Ca^2+^ uptake, suggesting that the MCU plays a critical role in adapting neuronal function to varying metabolic demands across different brain regions [89].

Intracellular Ca^2+^ signaling and SLC25 family are not complete strangers. Indeed, Ca^2+^-sensitive mitochondrial carriers (CaMCs) exist and are encoded by several genes, including *SLC25A12*, *SLC25A13*, *SLC25A23*, *SLC25A24* and *SLC25A25*. They share a common structure, with EF-hand domains at the N-terminus and a conserved mitochondrial carrier domain at the C-terminus. AGC1 is especially abundant in the brain, where it contributes to the Ca^2+^-regulated MAS that supports NADH transport and may help protect neurons during glutamate-induced excitotoxicity. Mice lacking AGC1 show growth retardation and problems with motor coordination. APC1-4 facilitate the exchange of ATP-Mg^2+^ and inorganic phosphate across the IMM supporting ATP hydrolysis and ΔΨ_m_ maintenance. Second, APC2 physically interacts with MCU and MICU1 enhancing mitochondrial Ca^2+^ uptake when overexpressed [90] (Figure 1).

Additionally, MAMs play roles in lipid metabolism and apoptosis regulation, processes that are essential for neuronal survival [91,92]. Disruption of ER-mitochondria interactions has been implicated in NDs, some of them characterized by prolonged mitochondrial Ca^2+^ overload which induce the mitochondrial permeability transition pore (mPTP) opening, leading to cell death [91,93].

As a consequence of this overview, impaired balance between fusion and fission together with SLC25 deficiency concurs in disrupting synaptic function and to neurodegenerative pathologies.

## 4. SLC25 Family and Mitochondria-Associated Dysfunctions in Neurodegenerative Diseases

### 4.1. Alzheimer’s Disease

AD is a progressive neurodegenerative disorder and the leading cause of dementia in the elderly population. Recently, the global number of incident cases was estimated at 7.24 million, with an age-standardized incidence rate of 94.99 per 100,000 individuals [94]. Clinically, AD is characterized by a gradual decline in cognitive function and memory. In its early stages, patients frequently experience spatial disorientation and difficulty retaining recently acquired information [95]. Principal pathological hallmarks of AD include amyloid-β (Aβ) plaques, neurofibrillary tangles (NFTs), gliosis and neuronal loss, often accompanied by cerebrovascular amyloidosis, neuroinflammation and profound synaptic alterations [96]. Mitochondria play a central role in AD pathogenesis and several SLC25 family members are known to be directly or indirectly involved.

#### 4.1.1. UCPs

Among all UCPs, UCP2, UCP4 and UCP5 are prominently expressed in the central nervous system (CNS) where they regulate cell metabolism and energy homeostasis [97]. Though they were thought to mediate mild mitochondrial uncoupling by dissipating electrochemical gradient, thereby limiting mitochondrial ROS production and protecting against oxidative stress, UCPs, except UCP1, are no longer regarded mainly as proton uncouplers. Our group identified UCP2 as C4 metabolite exporter [98], UCP4A in *Drosophila* as unidirectional aspartate transporter [99] and UCP5 as a counter-exchanger for inorganic anions and dicarboxylates, linking sulfur metabolism to redox control [100]. In this scenario, UCPs tightly regulate cell metabolism, energy balance and redox hemostasis. Because mitochondrial fusion, fission and mitophagy are tightly coupled to mitochondrial bioenergetics and redox status, UCP-mediated regulation of ΔΨ_m_ and ROS also influences mitochondrial dynamics [101]. In the context of NDs, mitochondria exhibit an excessive ΔΨ_m_-driven fission and fragmentation [102].

In AD, UCPs show a remarkable alteration in their expression. In human AD patients, UCP4 and UCP5 progressively declined in the frontal lobe of AD brains with increasing Braak stage, whereas UCP2 shows a marked reduction only in the advanced stages of the disease [103]. Conversely, in human primary hippocampal neurons exposed to different doses of Aβ, UCP2 was markedly elevated, along with lactate dehydrogenase (LDH) and nitric oxide (NO) levels, suggesting a protective function against oxidative stress [104]. Supporting this notion, treatment with Oleanolic acid (OA) reduced Aβ accumulation, apoptosis and ROS in N2a/APP695swe cells by upregulating mitochondrial UCP2, potentially via stanniocalcin-1 (STC-1) signaling [105].

Consistently, overexpression of STC-1 in AD mice improved cognition, reduced hippocampal apoptosis and alleviated neuroinflammation and oxidative stress, which subsequently inhibited ERK1/2 activation [106]. Moreover, the clock modulator Nobiletin enhanced cortical UCP2 and UCP4 expression in female APP/PS1 mice, with a strong reduction in cortical Aβ plaque deposition and attenuated the expression of AD-related genes (*APP*, *BACE1*, *APOE*). These results link circadian regulation to mitochondrial function and support UCPs as therapeutic targets in AD [107]. A marked downregulation of UCP2 and UCP4 in the temporal cortex of AD brains has been monitored also concomitant with upregulation of glia maturation factor (GMF), inducible nitric oxide synthase (iNOS) and NF-κB p65. These findings suggest that GMF-mediated suppression of UCPs may exacerbate AD pathogenesis by fostering chronic oxidative injury [108]. Notably, the loss of UCP activity could further promote mitochondrial fragmentation through sustained oxidative stress and excessive activation of fission mediators such as DRP1, thereby amplifying neuronal vulnerability [109]. For what concerns the genetics of this group of carriers and the disease, the variant rs9472817 in UCP4 significantly influences susceptibility to late-onset AD, with the C allele acting as an additive risk factor in both sporadic and familial cases [110].

In the 3xTg-AD transgenic mice model of AD, astrocytic UCP4 overexpression preserved dendritic arborization and counteracted metabolic alterations mainly related to the transport of C4 metabolites (i.e., L-aspartate) during the pathology, highlighting a therapeutic potential against AD and memory decline [111]. In the same model, age-related hypothermia and cold sensitivity occurred despite elevated UCP1; also, cold exposure worsened AD hallmarks [112]. Conversely, UCP1 deletion in the AD mice model Tg2576 induced sustained hyperthermia and significantly aggravated AD-like pathology, including increased Aβ, tau phosphorylation, glial activation and synaptic loss [113]. Collectively, these models establish that impaired thermoregulation, whether resulting in high or low body temperature, functions as a risk factor for accelerating AD neuropathology.

Panax notoginseng saponins (PNS), a plan extract of *Panax notoginseng* root, significantly upregulated UCP4 and UCP5 levels in the brain and prevented neuronal loss reducing 8-hydroxydeoxyguanosine production in SAMP8 AD-mouse model [114]. Again, housefly larvae (HL) powder protected APP/PS1 AD-mouse model from spatial memory deficits and hippocampal neuronal apoptosis, likely through antioxidant activity. HL treatment markedly reduced Aβ deposition, Cyclin D1 expression and promoted UCP4 upregulation. These neuroprotective effects were associated with activation of the P38 and JNK-MAPK signaling pathways [1]. Through these signaling axes, UCP-mediated improvements in metabolic restoration, redox balance and mQCM likely restore proper fission-fusion equilibrium, thereby preserving neuronal viability. Although not expressed in the brain, UCP1 appears to play a role in AD, or at least in some systemic symptoms associated with the condition. Indeed, plasma analysis from patients with AD, revealed increased expression of thioredoxin (Trx) accompanied by reduced expression of UCP1 compared with control subjects [115]. Altogether, these studies suggest that UCP2, UCP4 and UCP5 are downregulated in AD patients and various mouse models and their genetic or pharmacological restoration ameliorate the disease symptoms. The UCP1 seems to protect against AD through a general systemic mechanism involving the thermoregulation. Nevertheless, the protective role of UCPs in AD remains largely correlative conducted in diverse AD models with heterogeneous genetic backgrounds and further comprehensive mechanistic studies are required to link the metabolic transport function of UCPs to AD pathogenesis.

#### 4.1.2. ANT

ANT1 is the most abundant protein of the IMM and its dysregulation represents a key feature of mitochondrial dysfunction in NDs, particularly in AD. The first direct evidence linking ANT1 to AD was provided by Atlante et al., who showed that the neurotoxic NH2_26–44_ tau fragment impairs OXPHOS and depletes ATP by inhibiting ANT1 [116]. Building on this, Amadoro et al. demonstrated that the same tau fragment acts as a competitive inhibitor of ANT1, whereas Aβ_1–42_ impairs ANT1 function non-competitively, likely through an allosteric site [117].

Moreover, unlike full-length tau protein, the NH2-truncated fragment (aa 26–230) binds Aβ in AD synapses, forming a complex with ANT1 and cyclophilin D that disrupts mQCM and reduces mitochondrial mass [1]. Notably, extracellular ADP counteracts this toxicity by activating antioxidant defenses and fully preserving ANT1 activity, highlighting ADP and its derivatives as promising neuroprotective agents [118]. Finally, ANT1 upregulation alone is sufficient to induce mitochondrial dysfunction, including reduced mtDNA content and impaired Ca^2+^ buffering, by promoting mPTP opening. This process, driven by RCAN1-mediated stabilization of ANT1 mRNA, sensitizes neurons to apoptosis, whereas ANT1 silencing prevents cell death [119]. Genetic disruption of *SLC25A1* and *SLC25A4* induces severe mitochondrial dysfunction and markedly increases APOE expression and secretion [120]. ANT1 is included in a predictive model for early AD risk based on twelve glucose metabolism-related genes [121], and its rs7660552 variant has been specifically associated with racial disparities in Alzheimer’s incidence among older Black and White adults in the United States [122].

#### 4.1.3. MTCH1/SLC25A49/PSAP

In 1999, Xu et al. identified a novel protein, PSAP, which interacts with presenilin-1 (PS1), a key factor in AD pathogenesis [123]. Overexpression of PSAP was subsequently shown to induce apoptotic cell death through cytochrome c release from mitochondria [124]. This protein was later classified within the SLC25 family and officially designated as MTCH1. Notably, PSAP is often used as an alternative name for MTCH1; however, this abbreviation is potentially misleading because, according to the NCBI Gene database, PSAP is also the official gene symbol for Prosaposin. Since both MTCH1 and Prosaposin have been implicated in AD, this review uses PSAP exclusively to indicate the mitochondrial carrier MTCH1. According to Zeng et al., PS1-induced apoptosis operates through two pathways: a minor γ-secretase-dependent turnover of c-FLIP and a predominant γ-secretase–independent route. The latter is mediated by PSAP, which forms a PSAP-Bax complex upon PS1 stimulation. Knockdown of PSAP markedly suppresses this process, demonstrating its role as a critical mediator of Bax translocation to mitochondria [125]. Consistently, knockdown of PSAP markedly inhibited DR6-induced apoptosis, in line with its direct interaction with the death receptor. Mechanistically, PSAP mediates DR6-triggered cell death by preventing Bax translocation to mitochondria and the subsequent release of apoptotic factors, including cytochrome c, suggesting that it functions as an anchor or target for DR6-activated Bax [126]. Furthermore, PS1 mutations render cells more vulnerable to cellular stress by disrupting mitochondrial function and Ca^2+^ regulation. PS1-mutant cells showed pronounced mitochondrial alterations, including reduced ΔΨ_m_, impaired OXPHOS, increased ROS production and fragmented mitochondrial morphology. Additionally, these cells exhibited abnormal Ca^2+^ handling, with excessive mitochondrial Ca^2+^ uptake and defective buffering capacity, further aggravating mitochondrial dysfunction [127]. Together, these findings indicate that PS1 mutations compromise mitochondrial integrity, dynamics, and bioenergetics, sensitizing neurons to stress and contributing to the pathophysiology of AD.

In AD brains, PSAP is markedly expressed in the neocortex, where it shows a strong correlation with phosphorylated tau and a co-localization with neuronal and neuritic tangles, including ghost tangles. Collectively, these findings position PSAP as a pivotal mitochondrial carrier that integrates death receptor- and tau-associated apoptotic pathways, underscoring its potential contribution to selective neuronal vulnerability and AD progression.

#### 4.1.4. MTCH2/SLC25A50

MTCH2 is strongly associated with intrinsic cell death and mitochondrial metabolism. Accumulating evidence suggests that it plays a significant role in various human diseases, including AD. MTCH2 was first implicated in AD by Escott-Price et al. through gene-based GWAS analysis [128], and subsequent work demonstrated that the AD risk SNP rs7120548 is associated with reduced MTCH2 expression in human brain tissue [129]. Ruggiero A. et al. identified MTCH2 as a key regulator of hippocampal neuronal function. In mice, forebrain-specific MTCH2 deletion impaired motor coordination and hippocampus-dependent cognitive processes, including spatial memory and long-term potentiation. MTCH2-deficient hippocampal neurons showed reduced mitochondrial motility and Ca^2+^ uptake, highlighting its role in mitochondrial dynamics [16].

#### 4.1.5. SLC25A38

Zhang et al. identified SLC25A38, also known as appoptosin, as a novel pro-apoptotic protein whose upregulation in NDs contributes to neuronal death. It promotes intrinsic caspase-dependent apoptosis by regulating heme synthesis, an effect counteracted by membrane-anchored APP, which retains SLC25A38 in the cytosol and limits its mitochondrial translocation [130]. Consistently, SLC25A38 overexpression in SH-SY5Y cells induces intrinsic caspase-dependent apoptosis, while curcumin treatment attenuates these pathological effects [131]. Moreover, SLC25A38 also drives mitochondrial fragmentation independently of ROS or caspase activation by disrupting the interaction between MFN1 and MFN2, thereby reducing fusion activity. Importantly, downregulation of SLC25A38 prevents Aβ-induced mitochondrial fragmentation and neuronal death [132]. More recently, Zhang et al. revealed that oxidative stress increases SLC25A38 levels by inhibiting its proteasomal degradation, leading to neuronal apoptosis through the JNK–FoxO1 pathway. Conversely, SLC25A38 downregulation protects neurons from oxidative injury, reinforcing the view that targeting this protein could mitigate AD-related neurodegeneration [133]. Collectively, these findings converge to identify SLC25A38 as a promising therapeutic target for preventing neuronal loss in AD.

#### 4.1.6. Preliminary Associations with Other SLC25

Several weaker associations between AD and other members of the SLC25 family have also been reported. The mitochondrial glutamate carrier GC2 is upregulated in AD models and in specific human brain regions, where its expression correlates with clinicopathological features. In neurons, Aβ42 induces SLC25A18 expression, leading to mitochondrial dysfunction, loss of ΔΨ_m_ and oxidative stress. Silencing SLC25A18 mitigates these effects, establishing it as a novel mitochondrial carrier implicated in AD pathogenesis [134]. In AD brains, SLC25A6 [135] and SLC25A11 [136] show significant downregulation in the caudate nucleus and temporal cortex, respectively. Similarly, PiC is reduced in both intraventricular-streptozocin rats and AD patients [137].

SLC25A44 is highly expressed across multiple brain regions in post mortem AD samples and strongly correlates with Braak staging [138]. Sun et al. identified SLC25A21 as a potential AD risk gene using Generalized Multifactor Dimensionality Reduction (GMDR) analysis across four independent GWAS datasets [139]. Transcriptome-wide association study of AD hippocampus have identified several SLC25 genes (i.e., *SLC25A10, SLC25A17, SLC25A22*) as AD susceptibility factors, with SLC25A22 downregulation in glutamatergic neurons strongly linked to hippocampal atrophy and accelerated dementia progression, exceeding the effects of age or ApoE4 [140]. Finally, integrative bioinformatics and machine learning approaches proposed SLC25A20 [1] and SLC25A46 [141] as novel biomarkers for AD.

### 4.2. Parkinson’s Disease

PD is a progressive neurodegenerative movement disorder and represents the second most common neurodegenerative condition worldwide after AD, affecting over 8.5 million people globally [142]. Clinically, PD is characterized by motor symptoms such as resting tremor, bradykinesia, rigidity and postural instability, as well as a broad range of non-motor symptoms including cognitive impairment, sleep disturbances, mood disorders and autonomic dysfunction [143,144]. The disease is primarily associated with the degeneration of midbrain dopaminergic neurons in the substantia nigra pars compacta, resulting in dopamine depletion and the presence of eosinophilic inclusion bodies characterized by abnormal α-synuclein accumulation [145]. Mitochondrial dysfunction is a central hallmark of PD, linking bioenergetic failure, oxidative stress and impaired quality control pathways to the selective vulnerability of dopaminergic neurons. As in AD, mitochondrial carriers are critically implicated in PD; notably, UCPs and ANT1 regulate cellular energy flux and stress responses, while mitoferrins (SLC25A37 and SLC25A28) mediate mitochondrial iron homeostasis.

#### 4.2.1. UCPs

The first evidence linking UCPs to neuroprotection in PD was provided by Horvath and colleagues. In their study, short-term oral coenzyme Q10 administration to male African green monkeys prevented the 1-methyl-4-phenyl-1,2,3,6-tetrahydropyridine (MPTP)-induced loss of dopaminergic neurons in the substantia nigra. This effect was ascribed to enhanced mitochondrial uncoupling via UCP2 activation, which limited oxidative stress and preserved mitochondrial function [146]. Subsequent studies showed that UCP2 overexpression protects nigral dopaminergic neurons in mice, whereas its deletion increases neuronal vulnerability, reduces striatal dopamine turnover, diminishes tyrosine hydroxylase and dopamine transporter expression and causes locomotor impairments [147,148]. Similarly, UCP2 deficiency in astrocytes also promotes neuroinflammation through NLRP3 inflammasome activation [149], while in *Drosophila*, UCP2 overexpression preserves dopaminergic neurons, prevents dopamine depletion and mitigates locomotor deficits [150]. Also, PINK1-deficient PD models exhibit UCP2 downregulation, increasing susceptibility to oxidative stress and mitochondrial dysfunction [151]. Conversely, in leucine-rich repeat kinase 2 (LRRK2)-associated PD, the G2019S mutation triggers mitochondrial depolarization and ATP depletion via kinase-dependent upregulation of UCP2 and UCP4, defects reversible by pharmacological inhibition of UCPs [152]. Overall, UCP2 modulates mitochondrial integrity, ATP level, MMP, ROS homeostasis and neuronal survival, thereby mediating the neuroprotective effects of bioactive molecules such as ghrelin, leptin and hydrogen sulfide [153,154,155]. Mechanistically, UCP2 exports C4 TCA metabolic intermediates out of mitochondria contributing to decreased oxaloacetate level and maintains the ATP level. Also, UCP2 indirectly preserves mitochondrial dynamics by preventing excessive fission, supporting fusion and mitophagy, and maintaining a functional mitochondrial network in dopaminergic neurons.

In addition to UCP2, other uncoupling proteins such as UCP1, UCP4 and UCP5 also play roles in PD pathophysiology. Notably, protein deglycase 1 (DJ-1, also known as PARK7), which is linked to familial forms of PD, regulates the expression of UCP4 and UCP5 and supports neuronal survival. Loss of DJ-1 leads to downregulation of UCP4 and UCP5, increased mitochondrial protein oxidation, and heightened vulnerability of dopaminergic neurons [1]. Consistently, in 6-OHDA-lesioned rats, DJ-1 administration upregulates UCP4, UCP5 and SOD2, promoting neuronal survival [156]. In PINK1- or PARKIN-deficient *Drosophila*, overexpression of UCP4A mitigates mitochondrial dysfunction, locomotor deficits and muscle degeneration caused by loss of *pink1* or *parkin*, suggesting that enhancing UCP4A function can protect against mitochondrial dysfunction relevant to Parkinson’s disease [99,157]. These studies together suggest that UCP2, UCP4 and UCP5 are downregulated during PD pathogenesis in various disease models and this may contribute to metabolic shift from efficient mitochondrial oxidative phosphorylation to impaired bioenergetics characterized by complex I deficiency, reduced ATP production, and increased reliance on glycolysis [158]. Similarly, the downregulation of these transporters may contribute to dysregulated mitochondrial dynamics, oxidative stress, impaired calcium homeostasis, and increased neuronal vulnerability, ultimately exacerbating dopaminergic neurodegeneration [159].

Systemic UCP1 also contributes indirectly to PD phenotypes. DJ-1 deficiency increases UCP1 expression in BAT, enhancing energy expenditure, whereas DJ-1 overexpression suppresses UCP1 and favors adiposity [160]. In 6-OHDA rats, UCP1 upregulation correlates with increased thermogenesis and sympathetic activity, potentially contributing to PD-related weight loss [161]. In contrast, PINK1 deficiency reduces UCP1, causing BAT dysfunction and conversion to white-like adipocytes [162]. These studies suggest that PD-linked genes modulate UCP1 expression, hence thermogenesis and energy expenditure, ultimately contributing to non-motor metabolic phenotypes such as weight loss or adiposity in Parkinson’s disease.

Overall, UCPs appear to play a critical role in the pathogenesis of PD. UCP2, UCP4 and UCP5 are generally downregulated, whereas UCP1 shows variable regulation, being either upregulated or downregulated. However, current evidence linking UCPs to PD pathogenesis remains largely correlative and fragmentary, with limited mechanistic insight. Therefore, further investigation is warranted to elucidate the precise roles of these proteins in PD and the metabolic derangements associated with the disease.

#### 4.2.2. ANT1

Given its central function in mitochondrial energy metabolism, Hudson et al. investigated ANT1 and DNA polymerase subunit gamma (POLG1) as candidate genes in a family with autosomal dominant progressive external ophthalmoplegia (PEO) and Parkinsonism. No ANT1 mutations were detected, indicating that the phenotype in this family was attributable to POLG1 defects rather than ANT1 [163]. Similarly, other screenings in additional families with the same phenotype also failed to identify ANT1 mutations [164,165]. Conversely, the ANT1 p.V289M variant was shown to impair mitochondrial function and, together with POLG1 mutations, to cause encephalomyopathy with PEO and parkinsonism [166]. Ding et al. subsequently demonstrated that ANT1 is downregulated in MPTP-treated mice and 1-methyl-4-phenylpyridinium iodide (MPP^+^)-exposed cells. The data presented in his paper were not confirmed by other methods used to evaluate ANT1 expression. From his results, it appears that loss of ANT1 may occur only in the brainstem; more importantly, ANT1 is mainly associated with α-synuclein, forming complexes that promote neuronal injury. Then, overexpression of functional ANT1 alleviated MPP^+^-induced cytotoxicity [167]. Supporting evidence indicates that defective glycosylation of ANT1 in PD brains promotes aggregation in neuronal cells [168]. Finally, ANT1 enrichment has been shown to protect against oxidative stress and mPTP opening, whereas the p.N177A mutant exacerbates mitochondrial dysfunction, underscoring ANT1 as both a pathogenic factor and a potential therapeutic target in PD [169].

#### 4.2.3. Mitoferrin-2

Exposure to MPP^+^ increases transcription and translation of genes and proteins related to iron transport, including a significant dysregulation of Mitoferrin-2 in PD, exacerbating oxidative stress [170]. In *Drosophila*, mitoferrin overexpression alleviates PINK1 loss-of-function phenotypes by restoring mitochondrial iron availability and respiration [171]. Furthermore, PINK1 mutations disrupt iron homeostasis and alter the expression of both mitoferrin-1 and -2, further linking defective iron handling to PD pathogenesis.

#### 4.2.4. Preliminary Associations with Other SLC25

Limited evidence of PD association with other members of the SLC25 family have been reported.

Davison et al. showed that Parkin overexpression alters SLC25A5 in HEK293 cells [172]. Bitetto et al. described biallelic SLC25A46 mutations in patients with PD and optic atrophy, while Goldstein et al. reported a potentially pathogenic missense mutation in SLC25A44 within the GBA-370Rec PD risk haplotype [173,174]. Gialluisi et al. identified 28 deleterious variants in 26 candidate PD genes, including *SLC25A39* [175].

The transcriptomic analysis of post mortem caudate nucleus revealed altered gene expression, including *SLC25A6*, potentially affecting mitochondrial ADP/ATP transport [135]. Finally, SLC25A48 and SLC25A20 were proposed as potential PD biomarkers [176,177,178].

### 4.3. Amyotrophic Lateral Sclerosis

ALS is the most common adult-onset motor neuron degenerative disease, with an estimated incidence ranging from 0.26 to 23.46 cases per 100,000 person-years, and showing considerable regional variation worldwide [179]. ALS is traditionally defined as a progressive degeneration of upper and lower motor neurons, leading to muscle weakness, stiffness and spasms. However, it is increasingly recognized as a complex disorder that is often accompanied by cognitive and behavioral changes [180]. Its pathogenesis is equally intricate, driven by diverse cellular and molecular alterations that are categorized into four major groups: impaired RNA metabolism, altered protein homeostasis and autophagy, abnormalities in cytoskeletal organization and trafficking, mitochondrial dysfunction. To date, more than 40 genes have been implicated in ALS, with mutations in SOD1 being particularly common due to their detrimental impact on mitochondrial integrity [181]. Studies using SOD1-mutant models have further underscored the central role of mitochondria and mitochondrial carriers, with particular emphasis on the contributions of UCP3 and UCP2.

#### 4.3.1. UCPs

Early studies by Dupuis et al. reported selective upregulation of UCP3 in the gastrocnemius muscle of SOD1-G86R mice, as well as in muscle biopsies from sporadic ALS patients [182]. The UCP3 upregulation seen therein may play a key role in metabolic reprogramming of ALS muscle toward increased fatty acid oxidation and decreased glucose utilization recently demonstrated in several studies [183,184]. Supporting this notion, modulation of caloric intake by SOD1-G93A mice model of ALS upregulated the expression of UCP3 in the skeletal muscle suggesting that UCP3 play a metabolic role in ALS pathogenesis [185,186]. Cold acclimation similarly increased UCP3 in skeletal muscle of ALS mice, attenuating mitochondrial swelling in motor neurons by reducing interactions between VDAC and ANT1, core components of the mitochondrial permeability transition pore (mPTP) [187,188]. Noticeably, UCP3 expression is influenced by muscle type, disease stage and systemic factors. Smittkamp et al. reported reduced UCP3 in the extensor digitorum longus but not in the tongue of end-stage SOD1-G93A rats, suggesting tissue-specific regulation [189]. To further explore the contribution of muscle mitochondrial uncoupling to ALS, Dupuis and colleagues developed the MCK-UCP1 mouse model, in which UCP1 is overexpressed specifically in skeletal muscle. Mild uncoupling and hypermetabolism in this model destabilized neuromuscular junctions (NMJs), demonstrating that muscle-confined mitochondrial dysfunction is sufficient to drive motor neuron degeneration. Crossing MCK-UCP1 mice with SOD1-G86R mutants further showed that muscle UCP1 overexpression exacerbates ALS pathology [190].

UCP2 also plays a complex role in ALS pathogenesis. In SOD1-G93A mice, overexpression of hUCP2 reduced mitochondrial ROS, without noticeable uncoupling effect in G93A brain mitochondria, but accelerated disease progression, shortened survival and impaired mitochondrial Ca^2+^ uptake, suggesting that neuroprotective effect of hUCP2 is disease-specific and in the context of ALS it worsen the diseases progression likely via decline in mitochondrial Ca^2+^ uptake [191]. Inhibition of UCP2 with Genipin increased ATP production and restored mitochondrial morphology without affecting ROS, likely due to the fact that UCP2 functions as a metabolic gatekeeper, exporting C4 metabolites such as oxaloacetate to regulate acetyl-CoA utilization. Chronic reliance on fatty acid oxidation and ketogenesis may, however, exacerbate neuronal damage [192]. Similar to UCP3, UCP2 expression is modulated by ALS-associated stressors. In NSC-34 cells, transfection with wild-type or mutant TDP-43 increased UCP2 [193]. Consistently, UCP2 mRNA is elevated in hypoglossal motor neurons of end-stage SOD1-G93A mice [194], while PGC-1α silencing further suppresses UCP2 expression, worsening mitochondrial dysfunction [1]. Overall, UCP2 and UCP3 influence mitochondrial bioenergetics, Ca^2+^ handling and membrane potential, thereby modulating mitochondrial dynamics, including fusion/fission balance and mitophagy, which are critical for motor neuron survival in ALS. These pieces of evidence suggests that UCP2 and UCP3 play a key role in the onset of ALS and its progression, nevertheless, the results are usually inconsistent, warranting further studies to clarify their role more explicitly.

#### 4.3.2. Preliminary Associations with Other SLC25

Several minor associations between ALS and other members of the SLC25 family have been reported. Xu et al. showed that CoCl_2_-induced oxidative stress in NSC34 cells upregulated UCP4, reflecting an adaptive neuroprotective response [195]. Hadzhieva et al. reported that G93A-SOD1 SH-SY5Y cells exhibited increased expression of iron metabolism genes and elevated total cellular iron, while mitochondrial iron content remained unchanged despite upregulation of *SLC25A37* and *SLC25A28* (mitoferrin-1 and mitoferrin-2). These findings suggest an enhanced flux of iron into mitochondria [196]. Linseman et al. showed that OGC is extensively S-nitrosylated and functionally impaired in the spinal cord of SOD1-G93A mice, resulting in marked depletion of mitochondrial glutathione (GSH) and thereby contributing to ALS pathogenesis [197].

### 4.4. Multiple Sclerosis

MS is a complex, multifactorial chronic disorder of the central nervous system characterized by immune-mediated inflammation, demyelination, and neurodegeneration. Globally, the number of people affected reached 2.8 million in 2020, with an incidence rate of 2.1 per 100,000 [198]. Clinical manifestations vary according to demyelinating lesion location and commonly include optic neuritis, sensory and motor deficits, cerebellar and brainstem syndromes, fatigue, and cognitive or affective disturbances [199]. At the molecular level, MS pathogenesis involves autoreactive T and B cells that cross the blood–brain barrier, trigger pro-inflammatory cytokine cascades and promote demyelination. Microglia-derived oxidative stress drives mitochondrial dysfunction and neurodegeneration, with growing evidence implicating UCP2 as a key modulator.

#### 4.4.1. UCP2

The UCP2-866G/A promoter polymorphism, which reduces gene expression, was among the first findings linking UCP2 to multiple sclerosis (MS), showing association with increased disease susceptibility in German and Spanish cohorts [200,201]. Building on these findings, the role of UCP2 in MS was examined using an experimental autoimmune encephalomyelitis (EAE) mouse model. UCP2 knockout mice developed more severe disease than controls, with higher clinical scores, enhanced T- and B-cell responses, elevated pro-inflammatory cytokine production and increased ROS [202]. Interestingly, combined deficiency of iNOS and UCP2 reduced disease severity compared with iNOS knockout alone, suggesting a complex interplay between UCP2 and nitric oxide in modulating disease outcomes [203]. UCP2 expression was examined in the brain and spinal cord of mice at different stages of EAE, revealing specific upregulation in activated lymphocytes during neuroinflammation, likely reflecting a cellular attempt to counter oxidative stress and metabolic overload in rapidly proliferating immune cells [204]. Mechanistically, the export of C4 TCA intermediates by UCP2 can modulate mitochondrial metabolic flux and redox pressure, lowering mitochondrial ROS production and altering the balance between glycolytic and oxidative metabolism, processes deeply implicated in immune cell differentiation, activation and effector functions during MS pathology [205]. Beyond genetic and mechanistic links, therapeutic modulation of UCP2 expression has been implicated in MS and related neuroinflammatory contexts. Peroxisome proliferator-activated receptor-γ (PPAR-γ) agonists such as pioglitazone confer anti-inflammatory and neuroprotective effects in EAE, partly by preserving mitochondrial function and upregulating PGC-1α, UCP2 and COX1, thereby supporting oligodendrocyte precursor differentiation [206]. Similarly, Dang et al. demonstrated that overexpression of PGC-1α in EAE models and neuronal NSC-34 cells increases UCP2 (along with UCP4 and UCP5), implicating that the neuroprotective effect of PGC-1α is mediated at least partially through the restoration of uncoupling protein expression [207]. These studies suggest that UCP2 is downregulated during MS pathogenesis and its restoration could provide a neuroprotective effect against EAE and MS progression. Further studies are needed to investigate the mechanistic link between UCPs particularly UCP2 and MS progression.

#### 4.4.2. Preliminary Associations with Other SLC25

Several additional associations between MS and other members of the SLC25 family have been reported. Szolnoki et al. found that the rs10807344 CC variant of UCP4 exerted a protective effect against MS occurrence [208]. SLC25A37 (mitoferrin-1) is markedly upregulated in the peripheral blood mononuclear cells of MS patients [209], and variants have also been reported in two children with MS and iron deficiency, suggesting that impaired mitochondrial iron import may contribute to demyelination and disease pathogenesis [210].

### 4.5. Rare Neurodegenerative Diseases

Recent discoveries have revealed the importance of specific SLC25 transporters in the pathogenesis of rare neurodegenerative diseases. These often stem from genetic mutations that impair transporter function linking the metabolic disruption and mitochondrial impairments to progressive neuronal damage [11,211]. Unfortunately, research on biological samples from patients with (rare) NDs is challenging due to the limited number of diagnosed individuals and the difficulty in accessing well-preserved tissue or cells. These factors make it hard to collect sufficient, standardized and representative samples for meaningful analysis.

**Figure 2:**
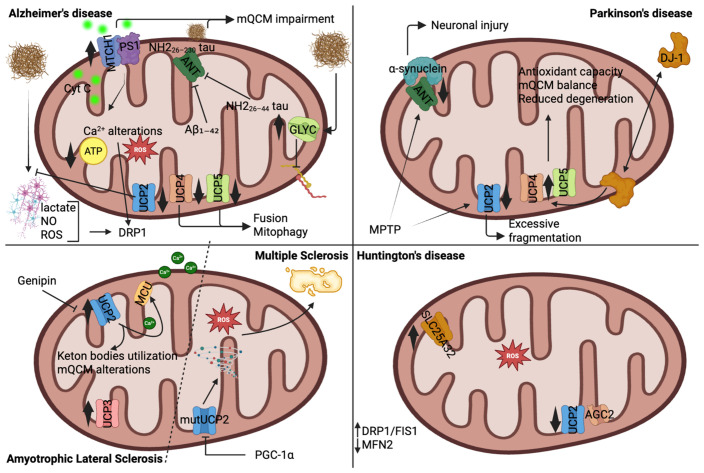
Mitochondrial carriers of the SLC25 family and their impact on mitochondrial dynamics in neurodegenerative diseases (NDs). For what concerns common NDs, the most described altered expressions of uncoupling proteins (UCP2, UCP4, UCP5) are reported; they disrupt mitochondrial membrane potential, ROS homeostasis and fusion–fission balance, contributing to neurodegeneration. Pathogenic interactions between Aβ, tau fragments and ANT1 (SLC25A4) impair ADP/ATP exchange, induce mitochondrial permeability transition and trigger apoptosis. MTCH1 (PSAP/SLC25A49) mediates presenilin-1-dependent Bax translocation and neuronal death, while the pro-apoptotic carrier SLC25A38 disrupts MFN1/MFN2 interaction, promoting fragmentation. For what concerns rare NDs, pathologic consequences of those diseases characterized by AGC1 (SLC25A12) and SLC25A46 deficiency have been depicted. Collectively, SLC25 dysfunction perturbs mitochondrial bioenergetics, dynamics and Ca^2+^ signaling, amplifying neuronal vulnerability. MPTP: 1-methyl-4-phenyl-1,2,3,6-tetrahydropyridine, mQCM: mitochondrial quality control mechanisms; NO: nitric oxide. Bolded black arrows: increased or decreased protein expression. Created with BioRender.com.

A rare syndrome with early-onset neurodegeneration is Leigh disease with optic atrophy and pontocerebellar hypoplasia. This pathology is part of a spectrum of disorders which have been described in mutations onset in charge of SLC25A46 [82,212,213]. Recessive mutations in SLC25A46 result in severe brain damage [214], strongly associated to the disruption of mitochondrial fission and cristae structure, impairing energy distribution and axonal integrity. In 2016, Janer A et al. identified a missense mutation (Thr142Ile) in a patient with classical Leigh syndrome. This mutation resulted in a significant reduction in SLC25A46 protein levels, suggesting a loss-of-function mechanism [82]. Interestingly, patient cells showed hyperfused mitochondria with an assembly defect in complex IV and reduced basal oxygen consumption [215]. The same results have been detected in zebrafish models of several optic atrophy spectrum disorders [84]. Associating mitochondrial hyperfusion to defects in respiration contradicts the physiological mechanisms at the basis of mitochondrial function expressed before. However, the presence of severely disorganized cristae can explain this phenotype. Indeed, a disruption of the MICOS complex has been reported, with the alteration of contact sites between IMM and OMM further negatively impacting on metabolites transport [215] (Figure 2).

SLC25A46 deficiency altered the morphology of the ER and reduced the number of mitochondria-ER contact sites, revealing a markedly altered phospholipid composition. Major classes such as cardiolipin, phosphatidylethanolamine, phosphatidylcholine, phosphatidylserine and phosphatidylglycerol were significantly changed in abundance compared to controls [82] (Figure 2). When wild-type SLC25A46 was reintroduced into deficient cells, both mitochondrial structure and respiratory function were partially restored, confirming the pathogenicity of the mutation [82].

Despite a second study supporting the findings of the previous report (a missense mutation, Leu341Pro and an exonic deletion/splice-site variant in SLC25A46 [214]), the role of SLC25A46 in mitochondrial dynamics is still far from being fully understood. More recently, a study using genetic screens in *C. elegans* identified SLC25A46 as a novel OMM fusion factor that would act upstream of MFNs. Thus, the absence of SLC25A46 would lead to markedly fragmented mitochondria [216], a morphological phenotype associated with accelerated neurodegeneration and compromised neuronal integrity. Overall, the work defines distinct and structurally validated interaction surfaces on SLC25A46 for both OPA1 and MFN2. These findings establish SLC25A46 as a molecular bridge that connects mitochondrial inner and outer membrane fusion machinery and clarify how mutations in SLC25A46 may contribute to mitochondrial dysfunction observed in certain neurodegenerative diseases [83]. However, it is not explained today how SLC25A46 deficiency can be associated with both fragmented and hyperfused mitochondria.

SLC25A46 has also been reported in an autosomal recessive form of ataxia [217], but no data about mitochondrial function have yet been described.

Huntington disease (HD) is a hereditary neurodegenerative condition inherited in an autosomal dominant manner. It results from an expanded CAG repeat in Exon 1 of the huntingtin gene, which produces a mutated form of the huntingtin protein. It disrupts the normal function and survival of neurons in the basal ganglia, causing a gradual decline in both motor skills and cognitive abilities.

A study modeling the human blood–brain barrier, established from differentiated iPS cells of HD donors, reported an altered expression of several *SLC25* genes with the 2-3-fold downregulation of *SLC25A8* and *SLC25A48* and a significant upregulation of *SLC25A13* and *SLC25A32* [218]. Belonging to the same family of genes, but investigated in platelets from HD patients through RNA-seq, also *SLC25A37*, *SLC25A39* and *SLC25A43* resulted to be differentially expressed when compared to healthy humans. Although this analysis should be further validated in vitro, considering the contribution of these carriers on mitochondrial function and considered the bioenergetics alterations found in those patients [219], their change in expression likely impacts mitochondrial metabolic dysfunction and impairment of the cerebrovascular vessel network investigated in this pool of patients. However, the findings obtained from the study of peripheral cells or the blood–brain barrier may not accurately reflect the scenario in the brain, even though these samples are from HD-affected patients. At the same time, few preclinical models are available, including transgenic R6/1 mice exhibiting a progressive neurological phenotype that mimics many of the features of HD. Here, researchers have not yet confirmed the differential expression of SLC25 transporters, but they found a compromised ability of HD astrocytes to clear glutamate, due to a reduced expression of plasma membrane transporter, potentially predispose neurons to excitotoxic damage. Concomitantly, these cells showed an upregulation of DRP1 coincided with a striking hypermetabolic phenotype: mitochondria in HD astrocytes exhibited increased basal and maximal oxygen consumption rates, higher ATP production, and elevated glycolytic activity as indicated by greater lactate secretion. Similar findings delineating an increased expression of DRP1 and FIS1 have been found in neurons [220] and decreased MFN2 [221]. However, this increased energy production came at a cost. Mitochondria of HD astrocytes also generated significantly more ROS, as evidenced by enhanced MitoSOX fluorescence. The accumulation of mitochondrial ROS likely contributes to oxidative stress within the astrocytes themselves and in the local environment. Impaired neuronal branching and dendritic complexity have been recorded [222] (Figure 2).

This is only one of the reasons explaining neuronal damage. Indeed, prolonged exposure to glutamate may disrupt intracellular Ca^2+^ signaling due to overexpression of NMDA receptors, with controversial effects at the mitochondrial level. In 2004, Khodorov B. summarized published studies highlighting issues related to increased mitochondrial Ca^2+^ uptake, mitochondrial dysfunction and mPTP opening-mediated cell death [223]. Conversely, another study conducted on the same transgenic R6/1 model attributed the problem to a reduced capacity to uptake and buffer cytosolic Ca^2+^ via MCU complex, resulting in prolonged and elevated cytosolic Ca^2+^ levels. The decreased mitochondrial Ca^2+^ handling capacity correlates with mitochondrial depolarization and impaired bioenergetic function [224]. This dysfunction compromises mitochondrial ATP production, which is necessary for Ca^2+^ extrusion mechanisms such as the plasma membrane Ca^2+^-ATPase (PMCA) and the sodium/calcium exchanger (NCX). These changes contribute to the generation of ROS and the initiation of apoptotic signaling cascades, ultimately promoting neurodegeneration [224].

It is remarkable how this scenario differently impacts various cellular populations.

Glutamate is extruded from astrocytes by SLC2A1, but it also needs to be transported into mitochondria for metabolism by SLC25A22. Deficits in glutamate transport in either direction are harmful: on one hand, they cause excitotoxic effects in the brain [222], and on the other hand, they impair mitochondrial glutamate metabolism. A study by Goubert E. and colleagues demonstrated a scenario of an in vitro model of rat astrocytes reducing SLC25A22 activity by gene silencing approaches decreases NAD(P)H production. The mitochondrial respiratory chain was not properly activated in response to glutamate and SLC25A22-silenced cells exhibited lower intracellular ATP levels compared to controls; also, there was an impairment of mitochondrial glutamate uptake. As a direct consequence, glutamate accumulates within the cytosol of astrocytes, thus disrupting the normal metabolic handling of glutamate, highlighting the tight coupling between mitochondrial function and glutamate homeostasis in astrocytes. Since astrocytes play a central role in regulating extracellular glutamate levels in the brain, impairment of mitochondrial glutamate transport may have important implications for conditions characterized by glutamate dysregulation and excitotoxicity [225].

## 5. Potential Therapeutic Strategies Targeting Mitochondria in Neurodegenerative Diseases

Targeting the above-mentioned mitochondrial dysfunctions and SLC25 carriers in NDs is an emerging field of research. While no therapies specifically target individual SLC25 or mitochondrial dynamic proteins clinically yet, several promising therapeutic strategies are being explored. These approaches aim to restore the function of the protein of interest, compensate for their loss or support overall mitochondrial health. They include: (i) gene therapy to restore functional copies of mutated genes; (ii) metabolic bypass and substrate supplementation by providing alternative metabolic fuels; (iii) mitochondrial biogenesis activators to enhance overall mitochondrial number and function to compensate for deficits and (iv) mitochondrial transfer.

### 5.1. Gene Therapy

This approach is preferentially suitable for those mitochondrial diseases with monogenic causes. Adeno-associated virus (AAV)-mediated delivery is one of the most widely used gene delivery systems, particularly in gene therapy. AAV have a good safety profile with low immunogenicity; they can lead to stable and long-term expression in neurons and muscle cells. The presence of different AAV serotypes allow the targeting of the gene of interest towards distinct tissues; however, their use is confined to only small inserts up to ~4.5 kb which do not always achieve the therapeutic expression requested in several tissues.

One of the most well-known examples of AAV-based therapy targets the SLC25A46 carrier, whose deficiency causes severe impairments in mitochondrial dynamics and neurodegeneration. In this study, researchers systemically administered an AAV-PHP.B vector carrying the wild-type *Slc25a46* gene to neonatal *Slc25a46^−/−^* mice. Treated animals showed significantly prolonged survival compared to untreated controls, which typically die within 5–8 weeks. Motor function was rescued, with improved locomotor activity and coordination on rotarod tests. At the mitochondrial level, therapy partially restored the activity of key respiratory chain complexes: Complex I in heart and skeletal muscle, Complex II+III in the brain, and Complex IV in heart and muscle, indicating functional recovery of mitochondrial bioenergetics in the most affected tissues [226]. Gene therapy has also been applied to other mitochondrial proteins. In Leigh syndrome (Complex I deficiency), AAV9-mediated delivery of human NDUFS4 corrected mitochondrial deficiencies and improved clinical outcomes in Ndufs4^−/−^ mice. IV or intracerebroventricular (ICV) administration alone restored Complex I activity in peripheral organs but did not improve lifespan. Combining IV and ICV administration enhanced Complex I activity, improved rotarod performance, and significantly prolonged survival, indicating partial amelioration of the clinical phenotype [226].

Therapies targeting mitochondrial dynamics have also been explored. In a mouse model of Dominant Optic Atrophy (DOA) caused by OPA1 mutations, AAV2-mediated delivery of human OPA1 cDNA into the vitreous chamber of Opa1^+/−^ mice restored mitochondrial and retinal function, highlighting the therapeutic potential of correcting mitochondrial fusion deficits [227]. Beyond SLC25A carriers and fusion/fission regulators, mitochondrial Ca^2+^ handling is emerging as a therapeutic target. In the hippocampus of the 3xTg AD mouse model, neuron-specific knockdown of MCU reduced mitochondrial Ca^2+^ uptake, leading to improvements in anxiety- and depressive-like behaviors without adverse effects. MCU silencing enhanced GAD67 expression, boosting GABA synthesis, and increased vGAT levels, a marker of inhibitory synaptic terminals. These changes suggest that excessive mitochondrial Ca^2+^ influx contributes to neuropsychiatric symptoms in AD and that targeting MCU can restore inhibitory neurotransmission [228]. Moreover, reducing MCU-mediated Ca^2+^ influx enhances the PKA-CREB-BDNF signaling pathway, which is critical for neuronal survival, synaptic plasticity, and antidepressant responses. Intracellular Ca^2+^ overload can degrade PKA via calpain activation, lowering CREB and BDNF levels, which are typically reduced in depression and AD. Knocking down MCU in hippocampal neurons restores PKA-CREB-BDNF signaling and produces antidepressant-like effects in AD models, further supporting mitochondrial Ca^2+^ regulation as a promising therapeutic strategy [229].

### 5.2. Metabolic Bypass and Substrate Supplementation

Metabolic bypass and substrate supplementation involves providing alternative substrates or extra amounts of metabolites, respectively, that can compensate for the loss or dysfunction of a specific transporter or enzyme.

AD is characterized by neuroinflammation and cellular senescence, mechanisms that involve activation of the cGAS–STING pathway by mtDNA released into the cytosol due to defective mitophagy [230]. AD neurons exhibit mitochondrial defects, including reduced mitochondrial biogenesis, impaired scavenging enzymes, and increased mitochondrial fragmentation driven by elevated DRP1 levels. These changes are accompanied by decreased expression of mitophagy-related proteins, such as phosphorylated TBK1 and ULK1, FUNDC1 and MUL1, which significantly impair oxygen consumption rate [231]. Similar findings have been observed in human studies [232]. Concomitantly, early amyloid plaques, gliosis, and cognitive deficits in AD mice correlate with a marked decline in the NAD^+^/NADH ratio compared to wild-type controls [233], a ratio that also declines with age [234]. Administration of nicotinamide riboside (NR), a precursor of NAD^+^, restores NAD^+^/NADH levels, induces mitophagy, reduces cytosolic mtDNA and inhibits cGAS–STING activation [233]. This results in decreased neuroinflammation, reduced cellular senescence and enhanced microglial clearance of Aβ in AD mouse models. NR-based strategies are currently being translated to clinical studies, including a phase I trial in Parkinson’s disease [235]. Notably, SLC25A51 is involved in NAD^+^ transport into mitochondria [53] and its loss would reduce mitochondrial NAD^+^ content and impairs respiration. Despite this strong link between SLC25A51, NAD^+^ and mitochondrial function, its role in AD pathogenesis remains unexplored. In rare NDs, SLC25A19 deficiency causes one of two phenotypes involving bilateral striatal degeneration and progressive polyneuropathy [236]. Biallelic mutations impair the mitochondrial thiamine carrier, compromising the activity of key metabolic enzymes, including the α-ketoglutarate dehydrogenase complex, and reducing cell viability [237,238]. Loss of SLC25A19 triggers the mitochondrial integrated stress response, marked by eIF2α phosphorylation and upregulation of ATF4 and DDIT4. Thiamine supplementation improves clinical outcomes and rescues mitochondrial dysfunction [239]. Similarly, Hyperornithinemia–hyperammonemia–homocitrullinuria (HHH) syndrome, caused by mutations in SLC25A15 (mitochondrial ornithine translocase), can result in progressive neurological impairment resembling neurodegenerative features. Treatment relies on substrate supplementation to bypass the metabolic block, restoring urea cycle function, reducing hyperammonemia, and supporting nitrogen excretion. Oral citrulline enters mitochondria independently of the ornithine transporter and is converted to argininosuccinate and then arginine, allowing the urea cycle to proceed. Arginine provides a downstream source of ornithine, maintaining nitrogen disposal even when mitochondrial ornithine uptake is impaired.

### 5.3. Mitochondrial Biogenesis Activators and Mitochondrial Dynamics Modulators

A plethora of findings link the modulation of mitochondrial shape to the improvement of the neurodegenerative phenotype. The modulation involves the use of small molecules, chaperones or drugs to preserve cristae architecture, the fusion/fission ratio, mitochondrial mass and damaged mitochondrial removal. These interventions are useful when gene therapy approaches are not available and can be dependent or not from SLC25 transporters activity.

#### 5.3.1. Targeting Mitochondrial Biogenesis in NDs

Pioglitazone, a peroxisome proliferator-activated receptor gamma (PPAR-γ) agonist is associated with a reduced risk of dementia in patients with both diabetes and a history of ischemic stroke. The authors conducted a large retrospective cohort study involving tens of thousands of individuals. The primary outcome assessed was the incidence of all-cause dementia over a follow-up period extending up to 15 years. The main finding of the study was that pioglitazone use was associated with a lower risk of developing dementia in patients with type 2 diabetes and prior ischemic stroke, compared to matched non-users. The authors proposed that the mechanism underlying this protective effect likely involves the anti-inflammatory and antioxidant properties of PPAR-γ activation [240].

Another study investigated the therapeutic potential of another PPAR agonist, bezafibrate in BACHD mice, a full-length transgenic model of Huntington’s disease (HD). HD is marked by mitochondrial dysfunction and oxidative stress, often due to impaired activity of PGC-1α, a master regulator of mitochondrial biogenesis and antioxidant defense. The authors demonstrated that bezafibrate effectively stimulates the PPAR–PGC-1α signaling pathway, restoring mitochondrial function and ameliorating both central and peripheral disease phenotypes. Bezafibrate treatment reversed PGC-1α pathway impairment in the striatum, soleus muscle and BAT, enhancing the expression of genes involved in mitochondrial biogenesis, such as NRF-1, cytochrome c and Tfam increasing mtDNA content and respiratory complex activity. This led to improvements in motor coordination, survival (28% increase) and neuropathological features in BACHD mice, including reduced neuronal loss, fewer pyknotic nuclei and normalized mitochondrial morphology. The treatment also mitigated oxidative stress by enhancing the expression of antioxidant genes (*HO-1, GR*) and restoring redox balance, as evidenced by normalized levels of MDA, 8-OHdG and the GSH/GSSG ratio in the brain. In skeletal muscle, bezafibrate reversed the pathological fiber-type switch from oxidative to glycolytic fibers and restored OXPHOS enzyme activity. In BAT, it reduced lipid accumulation and improved mitochondrial density and function, reversing hypothermic phenotypes characteristic of HD. Overall, the findings demonstrate that enhancing mitochondrial biogenesis via bezafibrate has neuroprotective and systemic benefits in HD. Given its long-standing clinical use and tolerability in treating hyperlipidemia, bezafibrate emerges as a promising candidate for repurposing in clinical trials for HD [241].

#### 5.3.2. Targeting Mitochondrial Fission in NDs

On the basis of evidence supporting abnormal mitochondrial dynamics as concauses of NDs, many authors targeted fission- and fusion-dependent proteins. Several molecules can be used to achieve this, like mitochondrial division inhibitor (Mdivi-1), a selective DRP1 inhibitor, that reversed mitochondrial dysfunction, synaptic failure and cognitive decline induced by Aβ in both neuronal cultures and in the transgenic mouse model APP/PS1. Also, oxidative markers were lowered. This study highlighted mitochondrial fission as a promising upstream therapeutic target in AD pathogenesis [242]. A second mitochondrial fission inhibitor, P110, which targets the binding between DRP1 and FIS1, prevented mitochondrial fragmentation in microglia and reduced the transfer of dysfunctional mitochondria which in turn mitigates the propagation of neuroinflammation and neuronal damage. Indeed, fragmented mitochondria interacted with neurons and astrocytes, leading to increased neuronal injury [1].

#### 5.3.3. Targeting Mitochondrial Fusion in NDs

Recently, a new compound was developed, 8015-P2, derived from piperine. This is a small molecule which activates MFN characterized by a favorable pharmacokinetic profile and acted in a stereospecific, MFN-dependent manner. In vitro, it successfully promoted mitochondrial fusion and restored motility in MFN2-deficient cells, as well as in fibroblasts and motor neurons derived from Charcot–Marie–Tooth disease type 2A (CMT2A) patients, a hereditary axonal peripheral neuropathy. The therapeutic potential of 8015-P2 was tested in the MFN2 T105M knock-in mice. Daily oral administration of the compound over a six-week period led to a complete reversal of both motor and sensory deficits. Treated mice showed normalized motor performance on the RotaRod, recovery of CMAP amplitudes, restoration of axonal mitochondrial morphology and trafficking, and reversal of neurogenic muscle atrophy and nerve degeneration. Notably, the therapeutic effects were reversible; discontinuation of treatment led to the gradual return of disease features, suggesting that continuous MFN activation is necessary to maintain neuronal and muscular health in this model [243].

Echinacoside (ECH), a phenylethanoid glycoside, has been explored for its neuroprotective properties, especially in neurodegenerative contexts like PD where mitochondrial Complex I deficiency is implicated. In experiments using in vitro approaches, researchers compared the ability of ECH to protect against cellular injury and mitochondrial dysfunction induced by inhibitors of respiratory chain complexes I through IV. Strikingly, ECH exhibited selective protection against damage caused by inhibitors of Complex I, cells exposed to MPP^+^ or rotenone retained mitochondrial membrane potential and avoided bioenergetic failure when treated with ECH. Further mechanistic investigation revealed that ECH’s neuroprotective effect stems from its ability to enhance the activity of mitochondrial Complex II. In the presence of Complex I inhibition, ECH increased Complex II-driven respiration, effectively bypassing the energy deficit caused by impaired Complex I and restoring cellular bioenergetics [244,245]. The rescue of mitochondrial respiratory impairment can also be the consequence of its described effect as fusion agonist and increased expression of MFN1 and MFN2 [246].

#### 5.3.4. Targeting Mitochondrial Mitophagy in NDs

In hippocampal tissue from Alzheimer’s patients, in iPSC-derived human AD neurons and in animal models of AD, the researchers found that mitophagy is significantly impaired, indicating an early defect in mQCM in the disease [230]. In an APP/PS1 transgenic mice, boosting mitophagy with Urolithin A (UA, and NAD^+^) reduced levels of insoluble Aβ_1–42_ and Aβ_1–40_, largely by activating microglial phagocytosis of extracellular plaques and suppressing neuroinflammation. Mice treated this way did not exhibit cognitive impairment, effectively preventing memory loss associated with Aβ pathology. Parallel experiments in tau-transgenic worms and mice showed that enhanced mitophagy completely abolished tau hyperphosphorylation in human neurons and reversed memory deficits in the animal models, demonstrating efficacy across both major AD pathological axes [230]. In cultured BV2 microglial cells exposed to LPS, UA treatment enhanced mitophagy, evidenced by increased colocalization of LC3 with mitochondria and elevated expression of mitophagy markers such as PINK1 and Parkin. This effect restored mitochondrial function and reduced ROS. Crucially, UA suppressed activation of the NLRP3 inflammasome, shown by lower levels of cleaved caspase-1, mature IL-1β and gasdermin D-mediated pyroptosis in these cells [247]. In vivo, in a MPTP-induced mouse model of PD, UA improved dopaminergic neuron survival and reversed behavioral deficits. It similarly inhibited microglial NLRP3 activation and neuroinflammation, effects that were directly linked to increased mitophagy. When mitophagy was disrupted, either pharmacologically or genetically, the neuroprotective and anti-inflammatory effects of UA were significantly diminished. Overall, the study supports a mechanism whereby UA induces microglial mitophagy, which in turn suppresses NLRP3 inflammasome activation and pyroptotic inflammation, leading to neuroprotection in Parkinson’s disease. It highlights microglial mitophagy enhancement as a promising therapeutic approach for PD by attenuating neuroinflammatory responses linked to mitochondrial dysfunction [247].

#### 5.3.5. Targeting Mitochondrial Cristae Remodeling in NDs

A study explored the therapeutic potential of SBT-272, a small molecule that stabilizes cardiolipin, a key phospholipid of the IMM, in the context of ALS. The authors used both in vitro and in vivo models to investigate how SBT-272 affects mitochondrial health linked to neuronal integrity in upper motor neurons (UMNs), which are known to be selectively vulnerable in ALS. SBT-272 was found to significantly improve mitochondrial structural integrity. This pathology often leads to fragmentation and swelling of mitochondria, impairing their capacity to produce ATP and maintain membrane potential. Treatment with SBT-272 restored the proper architecture of the IMM, likely by stabilizing cardiolipin and preserving cristae structure. These structural changes were accompanied by improvements in mitochondrial respiratory function, as shown by enhanced oxygen consumption and increased ATP production, both of which are crucial for maintaining neuronal viability. In chronic in vivo experiments, daily administration of SBT-272 to symptomatic mice resulted in a marked preservation of UMNs [248].

The inner membrane protein OPA1 is one of the main regulators of cristae remodeling [249]; recent advances in targeting OPA1 as a strategy to overcome cancer resistance [250,251,252,253] have led to the development of small molecules capable of modulating mitochondrial function, which may also be translatable to NDs.

### 5.4. Mitochondrial Transfer as Therapy Against NDs

Recent studies have demonstrated that functional mitochondria can be transferred from healthy donor cells, such as mesenchymal stem cells (MSCs), astrocytes, or even induced pluripotent stem cells (iPSCs) to damaged neurons via tunneling nanotubes, extracellular vesicles or gap junctions [1,254,255,256]. To the best of our knowledge, there are no reports focused on the SLC25 transporters about this matter, but such mitochondrial donation restores ATP production and rescues key aspects of neuronal metabolism, reducing oxidative stress and inhibiting apoptotic cascades [1,254,255,256]. So, mitochondrial transfer could potentially normalize SLC25 carrier function either directly, through the delivery of functional mitochondria expressing intact transporters, or indirectly, by relieving metabolic stress and restoring redox equilibrium. Importantly, the transferred mitochondria need to integrate efficiently into the host cell’s existing mitochondrial network, a process dependent on mitochondrial fusion, fission and trafficking mechanisms, which are themselves metabolically regulated [257,258].

Some researchers investigated how intravenous delivery of exogenous mitochondria might ameliorate Alzheimer’s-related pathology in a transgenic mouse model (5XFAD) [259]. In this study, the authors show that administering healthy mitochondria improves cognitive performance, reduces amyloid burden and rescues mitochondrial dysfunction in the brain. Interestingly, although the exogenously delivered mitochondria were detectable in the liver, they were not observed in the brain itself. Instead of a direct mitochondrial relocation into neurons, the authors propose a mechanism in which mitochondrial therapy triggers a cascade of metabolic and proteomic changes in the liver, which in turn alter levels of metabolites in the serum. These circulating metabolites then influence the brain’s environment in beneficial ways. They observed shifts in liver and serum metabolomics (e.g., in metabolites known to modulate neurodegenerative processes) and alterations in brain proteome linked to synaptic function, ubiquitin/proteasome pathways, phagocytosis and mitochondrial-related processes. A second study investigated the therapeutic potential of mitochondrial transfer in a mouse model in which aβ was injected intracerebroventricularly to induce Alzheimer-like pathology. The mice were treated with intravenous injections of freshly isolated, functional human mitochondria. Cognitive performance was assessed using behavioral tests, including the Y-maze and radial arm water maze, while neuropathological changes were evaluated through histological analysis. Mitochondrial function was also measured via enzyme activity assays, such as citrate synthase and cytochrome c oxidase [260]. Following treatment, the mitochondria-injected mice showed significant improvements in cognitive function compared to untreated Alzheimer’s mice, with performance in working memory and spatial learning tasks approaching that of healthy controls. Histological analysis revealed reduced neuronal loss and decreased gliosis in the hippocampus of treated mice. Furthermore, the activity of key mitochondrial enzymes in the brain was increased, indicating restored mitochondrial function. Notably, also in this case, enhanced mitochondrial activity was also observed in the liver, suggesting systemic effects of the therapy [260].

Thus, in the context of disorders associated with altered SLC25 carrier function, the delivery of healthy mitochondria could theoretically compensate for defects in metabolite transport, Ca^2+^ homeostasis and mitochondrial metabolism, thereby improving neuronal and glial resilience. This pathway might already occur among cells as a physiological mechanism of tissue repair, particularly through astrocyte–neuron communication, although in some severe conditions it cannot have therapeutic implications.

The exogenous administration of mitochondria can have several important limitations. The molecular mechanisms governing mitochondrial release, uptake, intracellular integration and long-term functionality remain incompletely understood. It is also unclear whether transferred mitochondria can stably integrate into endogenous mitochondrial networks and provide sustained benefits in chronic ND disorders, where pathogenic processes persist over many years. Additional challenges include the efficient and selective delivery of mitochondria to affected cell populations, potential barriers imposed by the blood–brain barrier, variability in mitochondrial quality between donors and the need for standardized protocols for mitochondrial isolation, storage and administration. Although autologous mitochondrial transplantation may reduce immunological concerns, the possibility of immune activation or unintended effects on cellular signaling cannot yet be excluded. Moreover, because many neurological disorders involve complex genetic and metabolic alterations, mitochondrial replacement alone may not fully correct the underlying disease mechanisms.

Therefore, while mitochondrial transfer represents a promising and innovative therapeutic avenue, its current application should be viewed with cautious optimism. Future studies should focus on defining the mechanisms regulating mitochondrial trafficking, identifying the most responsive target cell populations, establishing long-term safety profiles, and determining whether mitochondrial transfer can be effectively combined with approaches aimed at correcting the primary molecular defects, including those involving SLC25 carrier dysfunction.

## 6. Conclusions

NDs represent a multifaceted group of disorders characterized by the progressive loss of neuronal integrity, often rooted in mitochondrial dysfunction and disrupted cellular metabolism. The evidence summarized in this review underscores the central role of mitochondrial dynamics and the metabolic aspects of the SLC25 transporter family in maintaining neuronal homeostasis. Mitochondria are not static organelles but highly adaptable entities whose morphology, trafficking and metabolic output are finely tuned to meet the energetic and signaling demands of neurons. Dysregulation of these processes initiates a cascade of pathological events, including oxidative stress, calcium imbalance and bioenergetic collapse, which ultimately contribute to neuronal death.

The SLC25 family emerges as a pivotal component in this network, mediating the exchange of key metabolites and cofactors across the IMM. Mutations or altered expression of SLC25 genes disrupt mitochondrial communication with other cellular compartments, impairing energy metabolism and redox balance. Importantly, several members of this transporter family have now been linked to specific neurodegenerative phenotypes. These findings provide a mechanistic bridge between metabolic dysregulation and neurodegenerative pathology, positioning SLC25 carriers as both biomarkers and potential therapeutic targets. Therapeutically, understanding the dual relationship between mitochondrial dynamics and SLC25-mediated transport opens new perspectives for intervention. Modulating transporter activity, restoring mitochondrial morphology or enhancing biogenesis may counteract the early stages of neuronal decline. Furthermore, strategies such as antioxidant therapy, gene editing and small-molecule modulators targeting specific SLC25 carriers hold promise for personalized medicine approaches. Integrating these therapies with agents that stabilize mitochondrial fusion-fission balance or calcium homeostasis could yield synergistic benefits in halting or reversing disease progression.

Despite these advances, key challenges remain. The interplay between mitochondrial dynamics, SLC25 transport and neuronal survival is highly complex, necessitating deeper functional and structural characterization of individual transporters. Future research should aim to map transporter networks across neuronal subtypes and disease states, while leveraging high-resolution imaging and omics technologies to identify precise molecular signatures. Ultimately, decoding how SLC25 transporters coordinate with mitochondrial remodeling will be crucial for translating basic insights into effective clinical therapies. A more complete understanding of these processes may redefine the landscape of neurodegeneration research, offering tangible hope for preventing or mitigating mitochondrial-driven neuronal loss.

For what concerns one of the most promising therapies, like mitochondrial transfer, the compatibility of donor and recipient mitochondria in terms of mtDNA and nuclear-encoded mitochondrial proteins, including SLC25 transporters, remains an important consideration for the long-term success of this approach. Advances in bioengineering, such as the use of mitochondria-loaded nanocarriers or artificial mitochondrial transfer systems, may allow for more targeted delivery and functional assessment of transferred organelles. Moreover, coupling mitochondrial transfer with metabolic modulators or SLC25-targeted therapeutics may enhance efficacy by stabilizing mitochondrial function post-transfer. Despite encouraging preclinical data, challenges remain in scaling and translating these interventions to clinical use, including immunogenicity, delivery precision and mitochondrial viability during transfer. Nevertheless, integrating mitochondrial transfer strategies with a detailed understanding of mitochondrial metabolism and SLC25 transporter biology holds significant promise for addressing the metabolic underpinnings of neurodegeneration, potentially opening a new frontier in regenerative neurology.

## Figures and Tables

**Table 1:** Overview of identified human SLC25 family transporters. The table shows mitochondrial carriers listed by gene name (first column) and protein name (second column, if available), classified by their typology (third column) and main transported substrates (fourth column). The last column reports the cell types described in the literature and in this review where these carriers play a specific role in disease development. Blank cells in the ‘protein’ or ‘main substrates’ columns indicate that no name or transport information is available. CNS: central nervous system.

Overview of Identified Human SLC25 Family Transporters				
Mitochondrial Carrier Name	Protein	Classification	Main Substrates	Cell Types
*SLC25A1*	CIC, CTP	Carboxylates	Citrate, isocitrate, malate, phosphoenolpyruvate	Astrocytes
*SLC25A10*	DIC		Malate, phosphate, succinate, sulphate, thiosulphate, sulphite	Neurons
*SLC25A11*	OGC		2-oxoglutarate, malate, succinate	Astrocytes, neurons
*SLC25A21*	ODC		2-oxoadipate, 2-oxoglutarate	No CNS cell type specified
*SLC25A7*	UCP1, thermogenin	UCP	H^+^	Brown adipose tissue
*SLC25A8*	UCP2, UCPH		Aspartate, malate, phosphate, oxaloacetate, sulfate	Astrocytes, neurons, oligodendrocytes
*SLC25A9*	UCP3		Aspartate, malate, sulphate, phosphate	No CNS cell type specified
*SLC25A14*	UCP5, BMCP1		Sulfate, sulfite, thiosulfate, phosphate, dycarboxylates	Neurons
*SLC25A27*	UCP4		H^+^	Astrocytes, neurons
*SLC25A30*	UCP6, KMCP1		Sulfate, sulfite, thiosulfate, phosphate, dycarboxylates	No CNS cell type specified
*SLC25A4*	AAC1, ANT1	Nucleotides	ADP, ATP	No CNS cell type specified
*SLC25A5*	AAC2, ANT2		ADP, ATP	No CNS cell type specified
*SLC25A6*	AAC3, ANT3		ADP, ATP	No CNS cell type specified
*SLC25A17*	PMP34, CFNC		CoA, FAD, NAD^+^, dephospho-CoA, adenosine 3′,5′-diphosphate, FMN, AMP, ADP	No CNS cell type specified
*SLC25A19*	DNC, TPC, MUP1, MCPHA		Thiamine diphosphate, thiamine phosphate, (deoxy)nucleotides	No CNS cell type specified
*SLC25A23*	APC2, SCaMC-3		ATP, ATP-Mg^++^, phosphate, ADP, AMP	Neuronal
*SLC25A32*	MFT, MFTC		FAD, folate	No CNS cell type specified
*SLC25A43*				No CNS cell type specified
*SLC25A12*	AGC1, aralar	Amino acids	Aspartate, glutamate, cysteinsufinate	Neurons
*SLC25A13*	AGC2, citrin		Aspartate, glutamate, cysteinsulfinate	Human blood–brain barrier
*SLC25A15*	ORC1, ORNT1		Ornithine, citrulline, lysine, arginine	No CNS cell type specified
*SLC25A18*	GC2		Glutamate	Neurons
*SLC25A20*	CAC, CACT		Carnitine, acylcarnitine	No CNS cell type specified
*SLC25A22*	GC1		Glutamate	Neurons
*SLC25A38*	GLYC		Glycine	Neurons
*SLC25A44*			Valine, leucine	No CNS cell type specified
*SLC25A48*			Choline	No CNS cell type specified
*SLC25A3*	PiC, PHC, PTP	Ions	Phosphate	No CNS cell type specified
*SLC25A28*	Mfrn2, mitoferrin2		Fe^2+^	No CNS cell type specified
*SLC25A37*	Mfrn1, mitoferrin1		Fe^2+^	No CNS cell type specified
*SLC25A46*		Others		Broad neuronal requirement
*SLC25A49*	MTCH1			Neurons
*SLC25A50*	MTCH2			Neurons

## Data Availability

No new data were created or analyzed in this study. Data sharing is not applicable.

## References

[1] Wilson D.M., Cookson M.R., Van Den Bosch L., Zetterberg H., Holtzman D.M., Dewachter I. (2023). Hallmarks of Neurodegenerative Diseases. Cell.

[2] Lamptey R.N.L., Chaulagain B., Trivedi R., Gothwal A., Layek B., Singh J. (2022). A Review of the Common Neurodegenerative Disorders: Current Therapeutic Approaches and the Potential Role of Nanotherapeutics. Int. J. Mol. Sci..

[3] Nichols E., Steinmetz J.D., Vollset S.E., Fukutaki K., Chalek J., Abd-Allah F., Abdoli A., Abualhasan A., Abu-Gharbieh E., Akram T.T. (2022). Estimation of the Global Prevalence of Dementia in 2019 and Forecasted Prevalence in 2050: An Analysis for the Global Burden of Disease Study 2019. Lancet Public Health.

[4] Klemmensen M.M., Borrowman S.H., Pearce C., Pyles B., Chandra B. (2024). Mitochondrial Dysfunction in Neurodegenerative Disorders. Neurotherapeutics.

[5] Morciano G., Rimessi A., Patergnani S., Vitto V.A.M., Danese A., Kahsay A., Palumbo L., Bonora M., Wieckowski M.R., Giorgi C. (2022). Calcium Dysregulation in Heart Diseases: Targeting Calcium Channels to Achieve a Correct Calcium Homeostasis. Pharmacol. Res..

[6] Giorgi C., Marchi S., Simoes I.C.M., Ren Z., Morciano G., Perrone M., Patalas-Krawczyk P., Borchard S., Jędrak P., Pierzynowska K. (2018). Mitochondria and Reactive Oxygen Species in Aging and Age-Related Diseases. Int. Rev. Cell Mol. Biol..

[7] Marchi S., Patergnani S., Missiroli S., Morciano G., Rimessi A., Wieckowski M.R., Giorgi C., Pinton P. (2018). Mitochondrial and Endoplasmic Reticulum Calcium Homeostasis and Cell Death. Cell Calcium.

[8] Simoes I.C.M., Morciano G., Lebiedzinska-Arciszewska M., Aguiari G., Pinton P., Potes Y., Wieckowski M.R. (2020). The Mystery of Mitochondria-ER Contact Sites in Physiology and Pathology: A Cancer Perspective. Biochim. Biophys. Acta Mol. Basis Dis..

[9] Lacombe A., Scorrano L. (2024). The Interplay between Mitochondrial Dynamics and Autophagy: From a Key Homeostatic Mechanism to a Driver of Pathology. Semin. Cell Dev. Biol..

[10] Gyimesi G., Hediger M. (2020). Sequence Features of Mitochondrial Transporter Protein Families. Biomolecules.

[11] Palmieri F. (2014). Mitochondrial Transporters of the SLC25 Family and Associated Diseases: A Review. J. Inherit. Metab. Dis..

[12] Palmieri F., Scarcia P., Monné M. (2020). Diseases Caused by Mutations in Mitochondrial Carrier Genes *SLC25*: A Review. Biomolecules.

[13] Singh P., Suman S., Chandna S., Das T.K. (2009). Possible Role of Amyloid-Beta, Adenine Nucleotide Translocase and Cyclophilin-D Interaction in Mitochondrial Dysfunction of Alzheimer’s Disease. Bioinformation.

[14] Wan Y., Guo S., Zhen W., Xu L., Chen X., Liu F., Shen Y., Liu S., Hu L., Wang X. (2024). Structural Basis of Adenine Nucleotides Regulation and Neurodegenerative Pathology in ClC-3 Exchanger. Nat. Commun..

[15] Horowitz M.P., Greenamyre J.T. (2010). Mitochondrial Iron Metabolism and Its Role in Neurodegeneration. J. Alzheimers Dis..

[16] Ruggiero A., Aloni E., Korkotian E., Zaltsman Y., Oni-Biton E., Kuperman Y., Tsoory M., Shachnai L., Levin-Zaidman S., Brenner O. (2017). Loss of Forebrain MTCH2 Decreases Mitochondria Motility and Calcium Handling and Impairs Hippocampal-Dependent Cognitive Functions. Sci. Rep..

[17] Keogh M.J., Chinnery P.F. (2015). Mitochondrial DNA Mutations in Neurodegeneration. Biochim. Biophys. Acta.

[18] Cha M.-Y., Kim D.K., Mook-Jung I. (2015). The Role of Mitochondrial DNA Mutation on Neurodegenerative Diseases. Exp. Mol. Med..

[19] Howell N., Elson J.L., Chinnery P.F., Turnbull D.M. (2005). mtDNA Mutations and Common Neurodegenerative Disorders. Trends Genet..

[20] Zhunina O.A., Yabbarov N.G., Grechko A.V., Yet S.-F., Sobenin I.A., Orekhov A.N. (2020). Neurodegenerative Diseases Associated with Mitochondrial DNA Mutations. Curr. Pharm. Des..

[21] Wang Y., Zhao M., Xie H.-X., Yu H.-Y., Yang J.-S., Qie L.-X., Liu N.-H., Chen J.-Q., Yi Z.-J., Zhou T.-J. (2025). Mitochondria-Targeted Gene Delivery Using Fluorinated Lipid Nanoparticles to Alleviate Leber’s Hereditary Optic Neuropathy. Nat. Commun..

[22] Ng Y.S., Bindoff L.A., Gorman G.S., Klopstock T., Kornblum C., Mancuso M., McFarland R., Sue C.M., Suomalainen A., Taylor R.W. (2021). Mitochondrial Disease in Adults: Recent Advances and Future Promise. Lancet Neurol..

[23] Saraste M., Walker J.E. (1982). Internal Sequence Repeats and the Path of Polypeptide in Mitochondrial ADP/ATP Translocase. FEBS Lett..

[24] Palmieri F. (1994). Mitochondrial Carrier Proteins. FEBS Lett..

[25] Robinson A.J., Kunji E.R.S. (2006). Mitochondrial Carriers in the Cytoplasmic State Have a Common Substrate Binding Site. Proc. Natl. Acad. Sci. USA.

[26] Palmieri F., Monné M. (2016). Discoveries, Metabolic Roles and Diseases of Mitochondrial Carriers: A Review. Biochim. Biophys. Acta.

[27] Ruprecht J.J., Kunji E.R.S. (2020). The SLC25 Mitochondrial Carrier Family: Structure and Mechanism. Trends Biochem. Sci..

[28] Kunji E.R.S., Mavridou V., King M.S., Cimadamore-Werthein C., Jaiquel Baron S., Jones S.A., King A.C., Springett R., Chand D., Palmer S.M. (2025). The Peculiar Properties of Mitochondrial Carriers of the SLC25 Family. Biochem. J..

[29] Klingenberg M. (2008). The ADP and ATP Transport in Mitochondria and Its Carrier. Biochim. Biophys. Acta.

[30] Klingenberg M. (1992). Structure-Function of the ADP/ATP Carrier. Biochem. Soc. Trans..

[31] Fiermonte G., De Leonardis F., Todisco S., Palmieri L., Lasorsa F.M., Palmieri F. (2004). Identification of the Mitochondrial ATP-Mg/Pi Transporter. Bacterial Expression, Reconstitution, Functional Characterization, and Tissue Distribution. J. Biol. Chem..

[32] Palmieri L., Pardo B., Lasorsa F.M., del Arco A., Kobayashi K., Iijima M., Runswick M.J., Walker J.E., Saheki T., Satrústegui J. (2001). Citrin and Aralar1 Are Ca(2+)-Stimulated Aspartate/Glutamate Transporters in Mitochondria. EMBO J..

[33] del Arco A., Satrústegui J. (1998). Molecular Cloning of Aralar, a New Member of the Mitochondrial Carrier Superfamily That Binds Calcium and Is Present in Human Muscle and Brain. J. Biol. Chem..

[34] Profilo E., Peña-Altamira L.E., Corricelli M., Castegna A., Danese A., Agrimi G., Petralla S., Giannuzzi G., Porcelli V., Sbano L. (2017). Down-Regulation of the Mitochondrial Aspartate-Glutamate Carrier Isoform 1 AGC1 Inhibits Proliferation and N-Acetylaspartate Synthesis in Neuro2A Cells. Biochim. Biophys. Acta Mol. Basis Dis..

[35] Fiermonte G., Palmieri L., Todisco S., Agrimi G., Palmieri F., Walker J.E. (2002). Identification of the Mitochondrial Glutamate Transporter. Bacterial Expression, Reconstitution, Functional Characterization, and Tissue Distribution of Two Human Isoforms. J. Biol. Chem..

[36] Lunetti P., Damiano F., De Benedetto G., Siculella L., Pennetta A., Muto L., Paradies E., Marobbio C.M.T., Dolce V., Capobianco L. (2016). Characterization of Human and Yeast Mitochondrial Glycine Carriers with Implications for Heme Biosynthesis and Anemia. J. Biol. Chem..

[37] Fiermonte G., Dolce V., David L., Santorelli F.M., Dionisi-Vici C., Palmieri F., Walker J.E. (2003). The Mitochondrial Ornithine Transporter. Bacterial Expression, Reconstitution, Functional Characterization, and Tissue Distribution of Two Human Isoforms. J. Biol. Chem..

[38] Porcelli V., Longo A., Palmieri L., Closs E.I., Palmieri F. (2016). Asymmetric dimethylarginine is transported by the mitochondrial carrier SLC25A2. Amino Acids.

[39] Porcelli V., Fiermonte G., Longo A., Palmieri F. (2014). The Human Gene *SLC25A29*, of Solute Carrier Family 25, Encodes a Mitochondrial Transporter of Basic Amino Acids. J. Biol. Chem..

[40] Yoneshiro T., Wang Q., Tajima K., Matsushita M., Maki H., Igarashi K., Dai Z., White P.J., McGarrah R.W., Ilkayeva O.R. (2019). BCAA Catabolism in Brown Fat Controls Energy Homeostasis through SLC25A44. Nature.

[41] Dolce V., Fiermonte G., Runswick M.J., Palmieri F., Walker J.E. (2001). The Human Mitochondrial Deoxynucleotide Carrier and Its Role in the Toxicity of Nucleoside Antivirals. Proc. Natl. Acad. Sci. USA.

[42] Spaan A.N., Ijlst L., van Roermund C.W.T., Wijburg F.A., Wanders R.J.A., Waterham H.R. (2005). Identification of the Human Mitochondrial FAD Transporter and Its Potential Role in Multiple Acyl-CoA Dehydrogenase Deficiency. Mol. Genet. Metab..

[43] Fiermonte G., Paradies E., Todisco S., Marobbio C.M.T., Palmieri F. (2009). A Novel Member of Solute Carrier Family 25 (SLC25A42) Is a Transporter of Coenzyme A and Adenosine 3′,5′-Diphosphate in Human Mitochondria. J. Biol. Chem..

[44] Shaw G.C., Cope J.J., Li L., Corson K., Hersey C., Ackermann G.E., Gwynn B., Lambert A.J., Wingert R.A., Traver D. (2006). Mitoferrin Is Essential for Erythroid Iron Assimilation. Nature.

[45] Dolce V., Fiermonte G., Messina A., Palmieri F. (1991). Nucleotide Sequence of a Human Heart cDNA Encoding the Mitochondrial Phosphate Carrier. DNA Seq..

[46] Indiveri C., Iacobazzi V., Tonazzi A., Giangregorio N., Infantino V., Convertini P., Console L., Palmieri F. (2011). The mitochondrial carnitine/acylcarnitine carrier: Function, structure and physiopathology. Mol. Aspects Med..

[47] Mizuarai S., Miki S., Araki H., Takahashi K., Kotani H. (2005). Identification of Dicarboxylate Carrier Slc25a10 as Malate Transporter in de Novo Fatty Acid Synthesis. J. Biol. Chem..

[48] Palmieri F., Quagliariello E., Klingenberger M. (1972). Kinetics and Specificity of the Oxoglutarate Carrier in Rat-Liver Mitochondria. Eur. J. Biochem..

[49] Fiermonte G., Dolce V., Palmieri L., Ventura M., Runswick M.J., Palmieri F., Walker J.E. (2001). Identification of the Human Mitochondrial Oxodicarboxylate Carrier. Bacterial Expression, Reconstitution, Functional Characterization, Tissue Distribution, and Chromosomal Location. J. Biol. Chem..

[50] Majd H., King M.S., Smith A.C., Kunji E.R.S. (2018). Pathogenic mutations of the human mitochondrial citrate carrier SLC25A1 lead to impaired citrate export required for lipid, dolichol, ubiquinone and sterol synthesis. Biochim. Biophys. Acta Bioenerg..

[51] Laloi M., Klein M., Riesmeier J.W., Müller-Röber B., Fleury C., Bouillaud F., Ricquier D. (1997). A Plant Cold-Induced Uncoupling Protein. Nature.

[52] De Leonardis F., Ahmed A., Vozza A., Capobianco L., Riley C.L., Barile S.N., Di Molfetta D., Tiziani S., DiGiovanni J., Palmieri L. (2024). Human Mitochondrial Uncoupling Protein 3 Functions as a Metabolite Transporter. FEBS Lett..

[53] Luongo T.S., Eller J.M., Lu M.-J., Niere M., Raith F., Perry C., Bornstein M.R., Oliphint P., Wang L., McReynolds M.R. (2020). SLC25A51 Is a Mammalian Mitochondrial NAD+ Transporter. Nature.

[54] Zaltsman Y., Shachnai L., Yivgi-Ohana N., Schwarz M., Maryanovich M., Houtkooper R.H., Vaz F.M., De Leonardis F., Fiermonte G., Palmieri F. (2010). MTCH2/MIMP Is a Major Facilitator of tBID Recruitment to Mitochondria. Nat. Cell Biol..

[55] Haitina T., Lindblom J., Renström T., Fredriksson R. (2006). Fourteen Novel Human Members of Mitochondrial Solute Carrier Family 25 (SLC25) Widely Expressed in the Central Nervous System. Genomics.

[56] Yamamuro T., Katoh D., Silva G.M., Nishida H., Oikawa S., Higuchi Y., Wang D., Fujimoto M., Yoshida N., Li M. (2026). Mitochondrial Control of Glycerolipid Synthesis by a PEP Shuttle. Cell.

[57] Sobieski C., Fitzpatrick M.J., Mennerick S.J. (2017). Differential Presynaptic ATP Supply for Basal and High-Demand Transmission. J. Neurosci..

[58] Silver I.A., Erecińska M. (1997). Energetic Demands of the Na+/K+ ATPase in Mammalian Astrocytes. Glia.

[59] Martínez J., Marmisolle I., Tarallo D., Quijano C. (2020). Mitochondrial Bioenergetics and Dynamics in Secretion Processes. Front. Endocrinol..

[60] Wei Y., Miao Q., Zhang Q., Mao S., Li M., Xu X., Xia X., Wei K., Fan Y., Zheng X. (2023). Aerobic Glycolysis Is the Predominant Means of Glucose Metabolism in Neuronal Somata, Which Protects against Oxidative Damage. Nat. Neurosci..

[61] Pan R.-Y., He L., Zhang J., Liu X., Liao Y., Gao J., Liao Y., Yan Y., Li Q., Zhou X. (2022). Positive Feedback Regulation of Microglial Glucose Metabolism by Histone H4 Lysine 12 Lactylation in Alzheimer’s Disease. Cell Metab..

[62] Späte E., Zhou B., Sun T., Kusch K., Asadollahi E., Siems S.B., Depp C., Werner H.B., Saher G., Hirrlinger J. (2024). Downregulated Expression of Lactate Dehydrogenase in Adult Oligodendrocytes and Its Implication for the Transfer of Glycolysis Products to Axons. Glia.

[63] Kasischke K.A., Vishwasrao H.D., Fisher P.J., Zipfel W.R., Webb W.W. (2004). Neural Activity Triggers Neuronal Oxidative Metabolism Followed by Astrocytic Glycolysis. Science.

[64] Goyal M.S., Hawrylycz M., Miller J.A., Snyder A.Z., Raichle M.E. (2014). Aerobic Glycolysis in the Human Brain Is Associated with Development and Neotenous Gene Expression. Cell Metab..

[65] Uthayakumar B., Soliman H., Bragagnolo N.D., Cappelletto N.I.C., Lee C.Y., Geraghty B., Chen A.P., Perks W.J., Ma N., Heyn C. (2023). Age-Associated Change in Pyruvate Metabolism Investigated with Hyperpolarized 13 C-MRI of the Human Brain. Hum. Brain Mapp..

[66] Liu L., Zhang K., Sandoval H., Yamamoto S., Jaiswal M., Sanz E., Li Z., Hui J., Graham B.H., Quintana A. (2015). Glial Lipid Droplets and ROS Induced by Mitochondrial Defects Promote Neurodegeneration. Cell.

[67] Quintana-Cabrera R., Scorrano L. (2023). Determinants and Outcomes of Mitochondrial Dynamics. Mol. Cell.

[68] Iwata R., Casimir P., Vanderhaeghen P. (2020). Mitochondrial Dynamics in Postmitotic Cells Regulate Neurogenesis. Science.

[69] Kochan S.M.V., Malo M.C., Jevtic M., Jahn-Kelleter H.M., Wani G.A., Ndoci K., Pérez-Revuelta L., Gaedke F., Schäffner I., Lie D.C. (2024). Enhanced Mitochondrial Fusion during a Critical Period of Synaptic Plasticity in Adult-Born Neurons. Neuron.

[70] Haigh J.L., New L.E., Filippi B.M. (2020). Mitochondrial Dynamics in the Brain Are Associated with Feeding, Glucose Homeostasis, and Whole-Body Metabolism. Front Endocrinol..

[71] Ishikawa K., Yamamoto S., Hattori S., Nishimura N., Tani H., Mito T., Matsumoto H., Miyakawa T., Nakada K. (2019). Acquired Expression of Mutant Mitofusin 2 Causes Progressive Neurodegeneration and Abnormal Behavior. J. Neurosci..

[72] Del Bo R., Moggio M., Rango M., Bonato S., D’Angelo M.G., Ghezzi S., Airoldi G., Bassi M.T., Guglieri M., Napoli L. (2008). Mutated Mitofusin 2 Presents with Intrafamilial Variability and Brain Mitochondrial Dysfunction. Neurology.

[73] Carelli V., Musumeci O., Caporali L., Zanna C., La Morgia C., Del Dotto V., Porcelli A.M., Rugolo M., Valentino M.L., Iommarini L. (2015). Syndromic Parkinsonism and Dementia Associated with OPA1 Missense Mutations. Ann. Neurol..

[74] Vevea J.D., Chapman E.R. (2023). Mitofusin 2 Sustains the Axonal Mitochondrial Network to Support Presynaptic Ca^2+^ Homeostasis and the Synaptic Vesicle Cycle in Rat Hippocampal Axons. J. Neurosci..

[75] Patergnani S., Bonora M., Bouhamida E., Danese A., Marchi S., Morciano G., Previati M., Pedriali G., Rimessi A., Anania G. (2021). Methods to Monitor Mitophagy and Mitochondrial Quality: Implications in Cancer, Neurodegeneration, and Cardiovascular Diseases. Methods Mol. Biol..

[76] Patergnani S., Morciano G., Carinci M., Leo S., Pinton P., Rimessi A. (2022). The “Mitochondrial Stress Responses”: The “Dr. Jekyll and Mr. Hyde” of Neuronal Disorders. Neural Regen. Res..

[77] Toyama E.Q., Herzig S., Courchet J., Lewis T.L., Losón O.C., Hellberg K., Young N.P., Chen H., Polleux F., Chan D.C. (2016). Metabolism. AMP-Activated Protein Kinase Mediates Mitochondrial Fission in Response to Energy Stress. Science.

[78] Patten D.A., Wong J., Khacho M., Soubannier V., Mailloux R.J., Pilon-Larose K., MacLaurin J.G., Park D.S., McBride H.M., Trinkle-Mulcahy L. (2014). OPA1-Dependent Cristae Modulation Is Essential for Cellular Adaptation to Metabolic Demand. EMBO J..

[79] Murata D., Roy S., Lutsenko S., Iijima M., Sesaki H. (2024). Slc25a3-Dependent Copper Transport Controls Flickering-Induced Opa1 Processing for Mitochondrial Safeguard. Dev. Cell.

[80] Dolce V., Fiermonte G., Palmieri F. (1996). Tissue-Specific Expression of the Two Isoforms of the Mitochondrial Phosphate Carrier in Bovine Tissues. FEBS Lett..

[81] Boulet A., Vest K.E., Maynard M.K., Gammon M.G., Russell A.C., Mathews A.T., Cole S.E., Zhu X., Phillips C.B., Kwong J.Q. (2018). The Mammalian Phosphate Carrier SLC25A3 Is a Mitochondrial Copper Transporter Required for Cytochrome c Oxidase Biogenesis. J. Biol. Chem..

[82] Janer A., Prudent J., Paupe V., Fahiminiya S., Majewski J., Sgarioto N., Des Rosiers C., Forest A., Lin Z.-Y., Gingras A.-C. (2016). SLC25A46 Is Required for Mitochondrial Lipid Homeostasis and Cristae Maintenance and Is Responsible for Leigh Syndrome. EMBO Mol. Med..

[83] Boopathy S., Luce B.E., Lugo C.M., Hakim P., McDonald J., Kim H.L., Ponce J., Ueberheide B.M., Chao L.H. (2024). Identification of SLC25A46 Interaction Interfaces with Mitochondrial Membrane Fusogens Opa1 and Mfn2. J. Biol. Chem..

[84] Abrams A.J., Hufnagel R.B., Rebelo A., Zanna C., Patel N., Gonzalez M.A., Campeanu I.J., Griffin L.B., Groenewald S., Strickland A.V. (2015). Mutations in SLC25A46, Encoding a UGO1-like Protein, Cause an Optic Atrophy Spectrum Disorder. Nat. Genet..

[85] Cogliati S., Frezza C., Soriano M.E., Varanita T., Quintana-Cabrera R., Corrado M., Cipolat S., Costa V., Casarin A., Gomes L.C. (2013). Mitochondrial Cristae Shape Determines Respiratory Chain Supercomplexes Assembly and Respiratory Efficiency. Cell.

[86] Balsa E., Soustek M.S., Thomas A., Cogliati S., García-Poyatos C., Martín-García E., Jedrychowski M., Gygi S.P., Enriquez J.A., Puigserver P. (2019). ER and Nutrient Stress Promote Assembly of Respiratory Chain Supercomplexes through the PERK-eIF2α Axis. Mol. Cell.

[87] Wang L., Yang Z., Satoshi F., Prasanna X., Yan Z., Vihinen H., Chen Y., Zhao Y., He X., Bu Q. (2024). Membrane Remodeling by FAM92A1 during Brain Development Regulates Neuronal Morphology, Synaptic Function, and Cognition. Nat. Commun..

[88] Tröger J., Dahlhaus R., Bayrhammer A., Koch D., Kessels M.M., Qualmann B. (2025). Mitochondria Are Positioned at Dendritic Branch Induction Sites, a Process Requiring Rhotekin2 and Syndapin I. Nat. Commun..

[89] Groten C.J., MacVicar B.A. (2022). Mitochondrial Ca^2+^ Uptake by the MCU Facilitates Pyramidal Neuron Excitability and Metabolism during Action Potential Firing. Commun. Biol..

[90] Hoffman N.E., Chandramoorthy H.C., Shanmughapriya S., Zhang X.Q., Vallem S., Doonan P.J., Malliankaraman K., Guo S., Rajan S., Elrod J.W. (2014). SLC25A23 Augments Mitochondrial Ca^2+^ Uptake, Interacts with MCU, and Induces Oxidative Stress-Mediated Cell Death. Mol. Biol. Cell.

[91] De Vos K.J., Mórotz G.M., Stoica R., Tudor E.L., Lau K.-F., Ackerley S., Warley A., Shaw C.E., Miller C.C.J. (2012). VAPB Interacts with the Mitochondrial Protein PTPIP51 to Regulate Calcium Homeostasis. Hum. Mol. Genet..

[92] Gómez-Suaga P., Pérez-Nievas B.G., Glennon E.B., Lau D.H.W., Paillusson S., Mórotz G.M., Calì T., Pizzo P., Noble W., Miller C.C.J. (2019). The VAPB-PTPIP51 Endoplasmic Reticulum-Mitochondria Tethering Proteins Are Present in Neuronal Synapses and Regulate Synaptic Activity. Acta Neuropathol. Commun..

[93] Morciano G., Naumova N., Koprowski P., Valente S., Sardão V.A., Potes Y., Rimessi A., Wieckowski M.R., Oliveira P.J. (2021). The Mitochondrial Permeability Transition Pore: An Evolving Concept Critical for Cell Life and Death. Biol. Rev. Camb. Philos. Soc..

[94] Li X., Feng X., Sun X., Hou N., Han F., Liu Y. (2022). Global, Regional, and National Burden of Alzheimer’s Disease and Other Dementias, 1990–2019. Front. Aging Neurosci..

[95] Zhang J., Zhang Y., Wang J., Xia Y., Zhang J., Chen L. (2024). Recent Advances in Alzheimer’s Disease: Mechanisms, Clinical Trials and New Drug Development Strategies. Signal Transduct. Target. Ther..

[96] Guo T., Zhang D., Zeng Y., Huang T.Y., Xu H., Zhao Y. (2020). Molecular and Cellular Mechanisms Underlying the Pathogenesis of Alzheimer’s Disease. Mol. Neurodegener..

[97] Ramsden D.B., Ho P.W.-L., Ho J.W.-M., Liu H.-F., So D.H.-F., Tse H.-M., Chan K.-H., Ho S.-L. (2012). Human Neuronal Uncoupling Proteins 4 and 5 (UCP4 and UCP5): Structural Properties, Regulation, and Physiological Role in Protection against Oxidative Stress and Mitochondrial Dysfunction. Brain Behav..

[98] Vozza A., Parisi G., De Leonardis F., Lasorsa F.M., Castegna A., Amorese D., Marmo R., Calcagnile V.M., Palmieri L., Ricquier D. (2014). UCP2 Transports C4 Metabolites out of Mitochondria, Regulating Glucose and Glutamine Oxidation. Proc. Natl. Acad. Sci. USA.

[99] Lunetti P., Gorgoglione R., Curcio R., Marra F., Pignataro A., Vozza A., Riley C.L., Capobianco L., Palmieri L., Dolce V. (2022). Drosophila Melanogaster Uncoupling Protein-4A (UCP4A) Catalyzes a Unidirectional Transport of Aspartate. Int. J. Mol. Sci..

[100] Gorgoglione R., Porcelli V., Santoro A., Daddabbo L., Vozza A., Monné M., Di Noia M.A., Palmieri L., Fiermonte G., Palmieri F. (2019). The Human Uncoupling Proteins 5 and 6 (UCP5/SLC25A14 and UCP6/SLC25A30) Transport Sulfur Oxyanions, Phosphate and Dicarboxylates. Biochim. Biophys. Acta (BBA) -Bioenerg..

[101] Ježek P., Holendová B., Garlid K.D., Jabůrek M. (2018). Mitochondrial Uncoupling Proteins: Subtle Regulators of Cellular Redox Signaling. Antioxid. Redox Signal..

[102] Chen W., Zhao H., Li Y. (2023). Mitochondrial Dynamics in Health and Disease: Mechanisms and Potential Targets. Signal Transduct. Target. Ther..

[103] De La Monte S.M., Wands J.R. (2006). Molecular Indices of Oxidative Stress and Mitochondrial Dysfunction Occur Early and Often Progress with Severity of Alzheimer’s Disease. J. Alzheimer’s Dis..

[104] Jun Z., Ibrahim M.M., Dezheng G., Bo Y., Qiong W., Yuan Z. (2015). UCP2 Protects against Amyloid Beta Toxicity and Oxidative Stress in Primary Neuronal Culture. Biomed. Pharmacother..

[105] Guo Q., He J., Zhang H., Yao L., Li H. (2020). Oleanolic Acid Alleviates Oxidative Stress in Alzheimer’s Disease by Regulating Stanniocalcin-1 and Uncoupling Protein-2 Signalling. Clin. Exp. Pharma Physio.

[106] Wang P., Li X.-L., Cao Z.-H. (2021). STC1 Ameliorates Cognitive Impairment and Neuroinflammation of Alzheimer’s Disease Mice via Inhibition of ERK1/2 Pathway. Immunobiology.

[107] Kim E., Nohara K., Wirianto M., Escobedo G., Lim J.Y., Morales R., Yoo S.-H., Chen Z. (2021). Effects of the Clock Modulator Nobiletin on Circadian Rhythms and Pathophysiology in Female Mice of an Alzheimer’s Disease Model. Biomolecules.

[108] Thangavel R., Kempuraj D., Zaheer S., Raikwar S., Ahmed M.E., Selvakumar G.P., Iyer S.S., Zaheer A. (2017). Glia Maturation Factor and Mitochondrial Uncoupling Proteins 2 and 4 Expression in the Temporal Cortex of Alzheimer’s Disease Brain. Front. Aging Neurosci..

[109] Yang Q., Wang L., Liang Y., He Q., Sun Q., Luo J., Cao H., Fang Y., Zhou Y., Yang J. (2023). Loss of UCP2 Causes Mitochondrial Fragmentation by OMA1-Dependent Proteolytic Processing of OPA1 in Podocytes. FASEB J..

[110] Montesanto A., Crocco P., Anfossi M., Smirne N., Puccio G., Colao R., Maletta R., Passarino G., Bruni A.C., Rose G. (2016). The Genetic Variability of UCP4 Affects the Individual Susceptibility to Late-Onset Alzheimer’s Disease and Modifies the Disease’s Risk in APOE-*ε* 4 Carriers. J. Alzheimer’s Dis..

[111] Rosenberg N., Reva M., Binda F., Restivo L., Depierre P., Puyal J., Briquet M., Bernardinelli Y., Rocher A., Markram H. (2023). Overexpression of UCP4 in Astrocytic Mitochondria Prevents Multilevel Dysfunctions in a Mouse Model of Alzheimer’s Disease. Glia.

[112] Vandal M., White P.J., Tournissac M., Tremblay C., St-Amour I., Drouin-Ouellet J., Bousquet M., Traversy M.-T., Planel E., Marette A. (2016). Impaired Thermoregulation and Beneficial Effects of Thermoneutrality in the 3×Tg-AD Model of Alzheimer’s Disease. Neurobiol. Aging.

[113] Jung C.-G., Yamashita H., Kato R., Zhou C., Matsushita H., Takeuchi T., Abdelhamid M., Chen Y., Michikawa M. (2023). Deletion of UCP1 in Tg2576 Mice Increases Body Temperature and Exacerbates Alzheimer’s Disease-Related Pathologies. Int. J. Mol. Sci..

[114] Huang J.-L., Jing X., Tian X., Qin M.-C., Xu Z.-H., Wu D.-P., Zhong Z.-G. (2017). Neuroprotective Properties of *Panax notoginseng* Saponins via Preventing Oxidative Stress Injury in SAMP8 Mice. Evid.-Based Complement. Altern. Med..

[115] Cornelius C., Trovato Salinaro A., Scuto M., Fronte V., Cambria M.T., Pennisi M., Bella R., Milone P., Graziano A., Crupi R. (2013). Cellular Stress Response, Sirtuins and UCP Proteins in Alzheimer Disease: Role of Vitagenes. Immun. Ageing.

[116] Atlante A., Amadoro G., Bobba A., De Bari L., Corsetti V., Pappalardo G., Marra E., Calissano P., Passarella S. (2008). A Peptide Containing Residues 26–44 of Tau Protein Impairs Mitochondrial Oxidative Phosphorylation Acting at the Level of the Adenine Nucleotide Translocator. Biochim. Biophys. Acta (BBA)-Bioenerg..

[117] Amadoro G., Corsetti V., Atlante A., Florenzano F., Capsoni S., Bussani R., Mercanti D., Calissano P. (2012). Interaction between NH2-Tau Fragment and Aβ in Alzheimer’s Disease Mitochondria Contributes to the Synaptic Deterioration. Neurobiol. Aging.

[118] Bobba A., Amadoro G., Azzariti A., Pizzuto R., Atlante A. (2014). Extracellular ADP Prevents Neuronal Apoptosis via Activation of Cell Antioxidant Enzymes and Protection of Mitochondrial ANT-1. Biochim. Biophys. Acta (BBA)-Bioenerg..

[119] Jiang H., Zhang C., Tang Y., Zhao J., Wang T., Liu H., Sun X. (2017). The Regulator of Calcineurin 1 Increases Adenine Nucleotide Translocator 1 and Leads to Mitochondrial Dysfunctions. J. Neurochem..

[120] Wynne M.E., Ogunbona O., Lane A.R., Gokhale A., Zlatic S.A., Xu C., Wen Z., Duong D.M., Rayaprolu S., Ivanova A. (2023). APOE Expression and Secretion Are Modulated by Mitochondrial Dysfunction. eLife.

[121] Wang Y., Shen Z., Wu H., Yu Z., Wu X., Zhou L., Guo F. (2023). Identification of Genes Related to Glucose Metabolism and Analysis of the Immune Characteristics in Alzheimer’s Disease. Brain Res..

[122] Kolpakov S., Yashkin A., Ukraintseva S., Yashin A., Akushevich I. (2025). Genome-Related Mechanisms Contributing to Differences in Alzheimer’s Disease Incidence Between White and Black Older US Adults. J. Racial Ethn. Health Disparities.

[123] Xu X., Shi Y., Wu X., Gambetti P., Sui D., Cui M.-Z. (1999). Identification of a Novel PSD-95/Dlg/ZO-1 (PDZ)-like Protein Interacting with the C Terminus of Presenilin-1. J. Biol. Chem..

[124] Xu X., Shi Y., Gao W., Mao G., Zhao G., Agrawal S., Chisolm G.M., Sui D., Cui M.-Z. (2002). The Novel Presenilin-1-Associated Protein Is a Proapoptotic Mitochondrial Protein. J. Biol. Chem..

[125] Zeng L., Hu C., Zhang F., Xu D.C., Cui M.-Z., Xu X. (2015). Cellular FLICE-like Inhibitory Protein (c-FLIP) and PS1-Associated Protein (PSAP) Mediate Presenilin 1-Induced γ-Secretase-Dependent and -Independent Apoptosis, Respectively. J. Biol. Chem..

[126] Zhang J., Zhao Z.J., Fu X., Niu H., Hu C., Dong Y., Cui M.-Z., Zhang F., Zeng L., Xu X. (2020). Proapoptotic Mitochondrial Carrier Homolog Protein PSAP Mediates Death Receptor 6 Induced Apoptosis. J. Alzheimer’s Dis..

[127] Rojas-Charry L., Calero-Martinez S., Morganti C., Morciano G., Park K., Hagel C., Marciniak S.J., Glatzel M., Pinton P., Sepulveda-Falla D. (2020). Susceptibility to Cellular Stress in PS1 Mutant N2a Cells Is Associated with Mitochondrial Defects and Altered Calcium Homeostasis. Sci. Rep..

[128] Escott-Price V., Bellenguez C., Wang L.-S., Choi S.-H., Harold D., Jones L., Holmans P., Gerrish A., Vedernikov A., Richards A. (2014). Gene-Wide Analysis Detects Two New Susceptibility Genes for Alzheimer’s Disease. PLoS ONE.

[129] Karch C.M., Ezerskiy L.A., Bertelsen S., Goate A.M., Alzheimer’s Disease Genetics Consortium (ADGC) (2016). Alzheimer’s Disease Risk Polymorphisms Regulate Gene Expression in the *ZCWPW1* and the *CELF1* Loci. PLoS ONE.

[130] Zhang H., Zhang Y., Chen Y., Huang X., Zhou F., Wang W., Xian B., Zhang X., Masliah E., Chen Q. (2012). Appoptosin Is a Novel Pro-Apoptotic Protein and Mediates Cell Death in Neurodegeneration. J. Neurosci..

[131] Zheng K., Zhang J., Zhang C., Zhang Y., Chen X. (2015). Curcumin Inhibits Appoptosin-Induced Apoptosis via Upregulating Heme Oxygenase-1 Expression in SH-SY5Y Cells. Acta Pharmacol. Sin..

[132] Zhang C., Shi Z., Zhang L., Zhou Z., Zheng X., Liu G., Bu G., Fraser P.E., Xu H., Zhang Y. (2016). Appoptosin Interacts with Mitochondrial Outer-Membrane Fusion Proteins and Regulates Mitochondrial Morphology. J. Cell Sci..

[133] Zhang C., Tan Z., Xie Y., Zhao Y., Huang T.Y., Lu Z., Luo H., Can D., Xu H., Zhang Y. (2019). Appoptosin Mediates Lesions Induced by Oxidative Stress Through the JNK-FoxO1 Pathway. Front. Aging Neurosci..

[134] Song J.-Y., Jia Y., Han H., Yang X.-H., Zhang J., Zhang Q., Wang S.-S., Wang C.-Y., Chen L., Zhang M. (2024). Increased Expression of SLC25A18 Is Associated with Alzheimer’s Disease and Is Involved in Aβ42-Induced Mitochondrial Dysfunction and Apoptosis in Neuronal Cells. Mitochondrion.

[135] Cunningham A., Barrett E., Risch S., Lee P.H.U., Lee C., Moghekar A., Patra P., Shim J.W. (2025). NFκB1: A Common Biomarker Linking Alzheimer’s and Parkinson’s Disease Pathology. Front. Neurosci..

[136] Mukherjee S., Russell J.C., Carr D.T., Burgess J.D., Allen M., Serie D.J., Boehme K.L., Kauwe J.S.K., Naj A.C., Fardo D.W. (2017). Systems Biology Approach to Late-onset Alzheimer’s Disease Genome-wide Association Study Identifies Novel Candidate Genes Validated Using Brain Expression Data and *Caenorhabditis elegans* Experiments. Alzheimer’s Dement..

[137] Homolak J., Markovic P., Virag D., Knezovic A., Osmanovic Barilar J., Loncar A., Salkovic-Petrisic M., Babic Perhoc A. (2025). Species-Specific Sensitivity to Intracerebroventricular Streptozotocin in Rats and Mice Highlights Pathways and Proteins Relevant to Alzheimer’s Disease. J. Neural Transm..

[138] Archer D.B., Eissman J.M., Mukherjee S., Lee M.L., Choi S., Scollard P., Trittschuh E.H., Mez J.B., Bush W.S., Kunkle B.W. (2024). Longitudinal Change in Memory Performance as a Strong Endophenotype for Alzheimer’s Disease. Alzheimer’s Dement..

[139] Sun J., Song F., Wang J., Han G., Bai Z., Xie B., Feng X., Jia J., Duan Y., Lei H. (2014). Hidden Risk Genes with High-Order Intragenic Epistasis in Alzheimer’s Disease. J. Alzheimer’s Dis..

[140] Tian J., Jia K., Wang T., Guo L., Xuan Z., Michaelis E.K., Swerdlow R.H., Alzheimer’s Disease Neuroimaging Initiative, Du H. (2024). Hippocampal Transcriptome-Wide Association Study and Pathway Analysis of Mitochondrial Solute Carriers in Alzheimer’s Disease. Transl. Psychiatry.

[141] Sharma A., Dey P. (2021). A Machine Learning Approach to Unmask Novel Gene Signatures and Prediction of Alzheimer’s Disease within Different Brain Regions. Genomics.

[142] Ou Z., Pan J., Tang S., Duan D., Yu D., Nong H., Wang Z. (2021). Global Trends in the Incidence, Prevalence, and Years Lived with Disability of Parkinson’s Disease in 204 Countries/Territories from 1990 to 2019. Front. Public Health.

[143] Bloem B.R., Okun M.S., Klein C. (2021). Parkinson’s Disease. Lancet.

[144] Váradi C. (2020). Clinical Features of Parkinson’s Disease: The Evolution of Critical Symptoms. Biology.

[145] Henrich M.T., Oertel W.H., Surmeier D.J., Geibl F.F. (2023). Mitochondrial Dysfunction in Parkinson’s Disease—A Key Disease Hallmark with Therapeutic Potential. Mol. Neurodegener..

[146] Horvath T.L., Diano S., Leranth C., Garcia-Segura L.M., Cowley M.A., Shanabrough M., Elsworth J.D., Sotonyi P., Roth R.H., Dietrich E.H. (2003). Coenzyme Q Induces Nigral Mitochondrial Uncoupling and Prevents Dopamine Cell Loss in a Primate Model of Parkinson’s Disease. Endocrinology.

[147] Andrews Z.B., Horvath B., Barnstable C.J., Elseworth J., Yang L., Beal M.F., Roth R.H., Matthews R.T., Horvath T.L. (2005). Uncoupling Protein-2 Is Critical for Nigral Dopamine Cell Survival in a Mouse Model of Parkinson’s Disease. J. Neurosci..

[148] Conti B., Sugama S., Lucero J., Winsky-Sommerer R., Wirz S.A., Maher P., Andrews Z., Barr A.M., Morale M.C., Paneda C. (2005). Uncoupling Protein 2 Protects Dopaminergic Neurons from Acute 1,2,3,6-methyl-phenyl-tetrahydropyridine Toxicity. J. Neurochem..

[149] Lu M., Sun X.-L., Qiao C., Liu Y., Ding J.-H., Hu G. (2014). Uncoupling Protein 2 Deficiency Aggravates Astrocytic Endoplasmic Reticulum Stress and Nod-like Receptor Protein 3 Inflammasome Activation. Neurobiol. Aging.

[150] Islam R., Yang L., Sah M., Kannan K., Anamani D., Vijayan C., Kwok J., Cantino M.E., Beal M.F., Fridell Y.-W.C. (2012). A Neuroprotective Role of the Human Uncoupling Protein 2 (hUCP2) in a Drosophila Parkinson’s Disease Model. Neurobiol. Dis..

[151] Akundi R.S., Zhi L., Sullivan P.G., Büeler H. (2013). Shared and Cell Type-Specific Mitochondrial Defects and Metabolic Adaptations in Primary Cells from PINK1-Deficient Mice. Neurodegener. Dis..

[152] Papkovskaia T.D., Chau K.-Y., Inesta-Vaquera F., Papkovsky D.B., Healy D.G., Nishio K., Staddon J., Duchen M.R., Hardy J., Schapira A.H.V. (2012). G2019S Leucine-Rich Repeat Kinase 2 Causes Uncoupling Protein-Mediated Mitochondrial Depolarization. Human. Mol. Genet..

[153] Andrews Z.B., Erion D., Beiler R., Liu Z.-W., Abizaid A., Zigman J., Elsworth J.D., Savitt J.M., DiMarchi R., Tschöp M. (2009). Ghrelin Promotes and Protects Nigrostriatal Dopamine Function via a UCP2-Dependent Mitochondrial Mechanism. J. Neurosci..

[154] Ho P.W.-L., Liu H.-F., Ho J.W.-M., Zhang W.-Y., Chu A.C.-Y., Kwok K.H.-H., Ge X., Chan K.-H., Ramsden D.B., Ho S.-L. (2010). Mitochondrial Uncoupling Protein-2 (UCP2) Mediates Leptin Protection Against MPP+ Toxicity in Neuronal Cells. Neurotox. Res..

[155] Lu M., Zhao F.-F., Tang J.-J., Su C.-J., Fan Y., Ding J.-H., Bian J.-S., Hu G. (2012). The Neuroprotection of Hydrogen Sulfide Against MPTP-Induced Dopaminergic Neuron Degeneration Involves Uncoupling Protein 2 Rather Than ATP-Sensitive Potassium Channels. Antioxid. Redox Signal..

[156] Sun S.-Y., An C.-N., Pu X.-P. (2012). DJ-1 Protein Protects Dopaminergic Neurons against 6-OHDA/MG-132-Induced Neurotoxicity in Rats. Brain Res. Bull..

[157] Wu K., Liu J., Zhuang N., Wang T. (2014). UCP4A Protects against Mitochondrial Dysfunction and Degeneration in *Pink1*/*Parkin* Models of Parkinson’s Disease. FASEB J..

[158] Brunialti E., Villa A., Szego E.M., La Vitola P., Drago D., Pavlovic R., Fontana L., Tuna D., Panzeri A., Meda C. (2025). Metabolic Reprogramming and Altered ATP Content Impair Neuroprotective Functions of Microglia in β-Glucocerebrosidase Deficiency Models. J. Neuroinflamm..

[159] Kumar R., T A., Singothu S., Singh S.B., Bhandari V. (2022). Uncoupling Proteins as a Therapeutic Target for the Development of New Era Drugs against Neurodegenerative Disorder. Biomed. Pharmacother..

[160] Wu R., Liu X., Sun J., Chen H., Ma J., Dong M., Peng S., Wang J., Ding J., Li D. (2017). DJ-1 Maintains Energy and Glucose Homeostasis by Regulating the Function of Brown Adipose Tissue. Cell Discov..

[161] Wang W., Meng X., Yang C., Fang D., Wang X., An J., Zhang J., Wang L., Lu T., Ruan H.-B. (2017). Brown Adipose Tissue Activation in a Rat Model of Parkinson’s Disease. Am. J. Physiol.-Endocrinol. Metab..

[162] Ko M.S., Yun J.Y., Baek I.-J., Jang J.E., Hwang J.J., Lee S.E., Heo S.-H., Bader D.A., Lee C.-H., Han J. (2021). Mitophagy Deficiency Increases NLRP3 to Induce Brown Fat Dysfunction in Mice. Autophagy.

[163] Hudson G., Schaefer A.M., Taylor R.W., Tiangyou W., Gibson A., Venables G., Griffiths P., Burn D.J., Turnbull D.M., Chinnery P.F. (2007). Mutation of the Linker Region of the Polymerase γ-1 (*POLG1*Gene Associated with Progressive External Ophthalmoplegia and Parkinsonism. Arch. Neurol..

[164] Sato K., Yabe I., Yaguchi H., Nakano F., Kunieda Y., Saitoh S., Sasaki H. (2011). Genetic Analysis of Two Japanese Families with Progressive External Ophthalmoplegia and Parkinsonism. J. Neurol..

[165] Baloh R.H., Salavaggione E., Milbrandt J., Pestronk A. (2007). Familial Parkinsonism and Ophthalmoplegia from a Mutation in the Mitochondrial DNA Helicase Twinkle. Arch. Neurol..

[166] Galassi G., Lamantea E., Invernizzi F., Tavani F., Pisano I., Ferrero I., Palmieri L., Zeviani M. (2008). Additive Effects of POLG1 and ANT1 Mutations in a Complex Encephalomyopathy. Neuromuscul. Disord..

[167] Ding W., Qi M., Ma L., Xu X., Chen Y., Zhang W. (2021). ADP/ATP Translocase 1 Protects against an α-Synuclein-Associated Neuronal Cell Damage in Parkinson’s Disease Model. Cell Biosci..

[168] Zhang W., Liu J., Chen Q., Ding W., Li S., Ma L. (2022). Identification of ADP/ATP Translocase 1 as a Novel Glycoprotein and Its Association with Parkinson’s Disease. Neurochem. Res..

[169] Liu J., Ding W., Chen Q., Peng Y., Kong Y., Ma L., Zhang W. (2025). Adenine Nucleotide Translocase 1 Promotes Functional Integrity of Mitochondria via Activating DDIT3-CytC Pathway and Intensifying Actin Filament Structures. Mol. Neurobiol..

[170] Carroll C.B., Zeissler M.-L., Chadborn N., Gibson K., Williams G., Zajicek J.P., Morrison K.E., Hanemann C.O. (2011). Changes in Iron-Regulatory Gene Expression Occur in Human Cell Culture Models of Parkinson’s Disease. Neurochem. Int..

[171] Wan Z., Xu J., Huang Y., Zhai Y., Ma Z., Zhou B., Cao Z. (2020). Elevating Bioavailable Iron Levels in Mitochondria Suppresses the Defective Phenotypes Caused by PINK1 Loss-of-Function in Drosophila Melanogaster. Biochem. Biophys. Res. Commun..

[172] Davison E.J., Pennington K., Hung C., Peng J., Rafiq R., Ostareck-Lederer A., Ostareck D.H., Ardley H.C., Banks R.E., Robinson P.A. (2009). Proteomic Analysis of Increased Parkin Expression and Its Interactants Provides Evidence for a Role in Modulation of Mitochondrial Function. Proteomics.

[173] Bitetto G., Malaguti M.C., Ceravolo R., Monfrini E., Straniero L., Morini A., Di Giacopo R., Frosini D., Palermo G., Biella F. (2020). SLC25A46 Mutations in Patients with Parkinson’s Disease and Optic Atrophy. Park. Relat. Disord..

[174] Goldstein O., Gana-Weisz M., Attar R., Bar-Shira A., Lederkremer M., Shiner T., Thaler A., Mirelman A., Giladi N., Orr-Urtreger A. (2021). The GBA-370Rec Parkinson’s Disease Risk Haplotype Harbors a Potentially Pathogenic Variant in the Mitochondrial Gene *SLC25A44*. Mol. Genet. Metab..

[175] Gialluisi A., Reccia M.G., Modugno N., Nutile T., Lombardi A., Di Giovannantonio L.G., Pietracupa S., Ruggiero D., Scala S., Gambardella S. (2021). Identification of Sixteen Novel Candidate Genes for Late Onset Parkinson’s Disease. Mol. Neurodegener..

[176] Liu X., Cheng R., Verbitsky M., Kisselev S., Browne A., Mejia-Sanatana H., Louis E.D., Cote L.J., Andrews H., Waters C. (2011). Genome-Wide Association Study Identifies Candidate Genes for Parkinson’s Disease in an Ashkenazi Jewish Population. BMC Med. Genet..

[177] Khan A., Unlu G., Lin P., Liu Y., Kilic E., Kenny T.C., Birsoy K., Gamazon E.R. (2024). Metabolic gene function discovery platform GeneMAP identifies SLC25A48 as necessary for mitochondrial choline import. Nat. Genet..

[178] Verkerke A.R.P., Shi X., Li M., Higuchi Y., Yamamuro T., Katoh D., Nishida H., Auger C., Abe I., Gerszten R.E. (2024). SLC25A48 controls mitochondrial choline import and metabolism. Cell Metab..

[179] Wolfson C., Gauvin D.E., Ishola F., Oskoui M. (2023). Global Prevalence and Incidence of Amyotrophic Lateral Sclerosis: A Systematic Review. Neurology.

[180] Feldman E.L., Goutman S.A., Petri S., Mazzini L., Savelieff M.G., Shaw P.J., Sobue G. (2022). Amyotrophic Lateral Sclerosis. Lancet.

[181] Nguyen L. (2024). Updates on Disease Mechanisms and Therapeutics for Amyotrophic Lateral Sclerosis. Cells.

[182] Dupuis L., Scala F., Rene F., Tapia M., Oudart H., Pradat P.-F., Meininger V., Loeffler J.-P. (2003). Up-regulation of Mitochondrial Uncoupling Protein 3 Reveals an Early Muscular Metabolic Defect in Amyotrophic Lateral Sclerosis. FASEB J..

[183] Steyn F.J., Li R., Kirk S.E., Tefera T.W., Xie T.Y., Tracey T.J., Kelk D., Wimberger E., Garton F.C., Roberts L. (2020). Altered Skeletal Muscle Glucose-Fatty Acid Flux in Amyotrophic Lateral Sclerosis. Brain Commun..

[184] Palamiuc L., Schlagowski A., Ngo S.T., Vernay A., Dirrig-Grosch S., Henriques A., Boutillier A.-L., Zoll J., Echaniz-Laguna A., Loeffler J.-P. (2015). A Metabolic Switch toward Lipid Use in Glycolytic Muscle Is an Early Pathologic Event in a Mouse Model of Amyotrophic Lateral Sclerosis. EMBO Mol. Med..

[185] Patel B.P., Safdar A., Raha S., Tarnopolsky M.A., Hamadeh M.J. (2010). Caloric Restriction Shortens Lifespan through an Increase in Lipid Peroxidation, Inflammation and Apoptosis in the G93A Mouse, an Animal Model of ALS. PLoS ONE.

[186] Golko-Perez S., Amit T., Bar-Am O., Youdim M.B.H., Weinreb O. (2017). A Novel Iron Chelator-Radical Scavenger Ameliorates Motor Dysfunction and Improves Life Span and Mitochondrial Biogenesis in SOD1G93A ALS Mice. Neurotox. Res..

[187] Martin L.J., Niedzwiecki M.V., Wong M. (2021). Chronic Intermittent Mild Whole-Body Hypothermia Is Therapeutic in a Mouse Model of ALS. Cells.

[188] Campo G., Morciano G., Pavasini R., Bonora M., Sbano L., Biscaglia S., Bovolenta M., Pinotti M., Punzetti S., Rizzo P. (2016). Fo ATP Synthase C Subunit Serum Levels in Patients with ST-Segment Elevation Myocardial Infarction: Preliminary Findings. Int. J. Cardiol..

[189] Smittkamp S.E., Spalding H.N., Brown J.W., Gupte A.A., Chen J., Nishimune H., Geiger P.C., Stanford J.A. (2010). Measures of Bulbar and Spinal Motor Function, Muscle Innervation, and Mitochondrial Function in ALS Rats. Behav. Brain Res..

[190] Dupuis L., Gonzalez De Aguilar J.-L., Echaniz-Laguna A., Eschbach J., Rene F., Oudart H., Halter B., Huze C., Schaeffer L., Bouillaud F. (2009). Muscle Mitochondrial Uncoupling Dismantles Neuromuscular Junction and Triggers Distal Degeneration of Motor Neurons. PLoS ONE.

[191] Peixoto P.M., Kim H.-J., Sider B., Starkov A., Horvath T.L., Manfredi G. (2013). UCP2 Overexpression Worsens Mitochondrial Dysfunction and Accelerates Disease Progression in a Mouse Model of Amyotrophic Lateral Sclerosis. Mol. Cell. Neurosci..

[192] Szelechowski M., Amoedo N., Obre E., Léger C., Allard L., Bonneu M., Claverol S., Lacombe D., Oliet S., Chevallier S. (2018). Metabolic Reprogramming in Amyotrophic Lateral Sclerosis. Sci. Rep..

[193] Lu J., Duan W., Guo Y., Jiang H., Li Z., Huang J., Hong K., Li C. (2012). Mitochondrial Dysfunction in Human TDP-43 Transfected NSC34 Cell Lines and the Protective Effect of Dimethoxy Curcumin. Brain Res. Bull..

[194] MÃ¼hling T., Duda J., Weishaupt J.H., Ludolph A.C., Liss B. (2014). Elevated mRNA-Levels of Distinct Mitochondrial and Plasma Membrane Ca^2+^ Transporters in Individual Hypoglossal Motor Neurons of Endstage SOD1 Transgenic Mice. Front. Cell. Neurosci..

[195] Xu R., Wu C., Zhang X., Zhang Q., Yang Y., Yi J., Yang R., Tao Y. (2011). Linking Hypoxic and Oxidative Insults to Cell Death Mechanisms in Models of ALS. Brain Res..

[196] Hadzhieva M., Kirches E., Wilisch-Neumann A., Pachow D., Wallesch M., Schoenfeld P., Paege I., Vielhaber S., Petri S., Keilhoff G. (2013). Dysregulation of Iron Protein Expression in the G93A Model of Amyotrophic Lateral Sclerosis. Neuroscience.

[197] Linseman D.A., Winter A.N., Wilkins H.M. (2022). The 2-Oxoglutarate Carrier Is S-Nitrosylated in the Spinal Cord of G93A Mutant hSOD1 Mice Resulting in Disruption of Mitochondrial Glutathione Transport. Biomedicines.

[198] Walton C., King R., Rechtman L., Kaye W., Leray E., Marrie R.A., Robertson N., La Rocca N., Uitdehaag B., Van Der Mei I. (2020). Rising Prevalence of Multiple Sclerosis Worldwide: Insights from the Atlas of MS, Third Edition. Mult. Scler..

[199] Filippi M., Bar-Or A., Piehl F., Preziosa P., Solari A., Vukusic S., Rocca M.A. (2018). Multiple Sclerosis. Nat. Rev. Dis. Prim..

[200] Vogler S., Goedde R., Miterski B., Gold R., Kroner A., Koczan D., Zettl U.-K., Rieckmann P., Epplen J.T., Ibrahim S.M. (2005). Association of a Common Polymorphism in the Promoter of UCP2 with Susceptibility to Multiple Sclerosis. J. Mol. Med..

[201] Otaegui D., Saenz A., Ruίz-Martίnez J., Olaskoaga J., López De Munain A. (2007). UCP2 and Mitochondrial Haplogroups as a Multiple Sclerosis Risk Factor. Mult. Scler..

[202] Vogler S., Pahnke J., Rousset S., Ricquier D., Moch H., Miroux B., Ibrahim S.M. (2006). Uncoupling Protein 2 Has Protective Function during Experimental Autoimmune Encephalomyelitis. Am. J. Pathol..

[203] Aheng C., Ly N., Kelly M., Ibrahim S., Ricquier D., Alves-Guerra M.-C., Miroux B. (2011). Deletion of UCP2 in iNOS Deficient Mice Reduces the Severity of the Disease during Experimental Autoimmune Encephalomyelitis. PLoS ONE.

[204] Smorodchenko A., Schneider S., Rupprecht A., Hilse K., Sasgary S., Zeitz U., Erben R.G., Pohl E.E. (2017). UCP2 Up-Regulation within the Course of Autoimmune Encephalomyelitis Correlates with T-Lymphocyte Activation. Biochim. Biophys. Acta (BBA)-Mol. Basis Dis..

[205] Chaudhuri L., Srivastava R.K., Kos F., Shrikant P.A. (2016). Uncoupling Protein 2 Regulates Metabolic Reprogramming and Fate of Antigen-Stimulated CD8+ T Cells. Cancer Immunol. Immunother..

[206] De Nuccio C., Bernardo A., Cruciani C., De Simone R., Visentin S., Minghetti L. (2015). Peroxisome Proliferator Activated Receptor-γ Agonists Protect Oligodendrocyte Progenitors against Tumor Necrosis Factor-Alpha-Induced Damage: Effects on Mitochondrial Functions and Differentiation. Exp. Neurol..

[207] Dang C., Han B., Li Q., Han R., Hao J. (2019). Up-regulation of PGC-1α in Neurons Protects against Experimental Autoimmune Encephalomyelitis. FASEB J..

[208] Szolnoki Z., Kondacs A., Mandi Y., Bodor A., Somogyvari F. (2009). A Homozygous Genetic Variant of Mitochondrial Uncoupling Protein 4 Exerts Protection Against the Occurrence of Multiple Sclerosis. Neuromol Med..

[209] Derakhshani A., Safarpour H., Abdoli Shadbad M., Hemmat N., Leone P., Asadzadeh Z., Pashazadeh M., Baradaran B., Racanelli V. (2021). The Role of Hemoglobin Subunit Delta in the Immunopathy of Multiple Sclerosis: Mitochondria Matters. Front. Immunol..

[210] Van Rensburg S.J., Peeters A.V., Van Toorn R., Schoeman J., Moremi K.E., Van Heerden C.J., Kotze M.J. (2019). Identification of an Iron-Responsive Subtype in Two Children Diagnosed with Relapsing-Remitting Multiple Sclerosis Using Whole Exome Sequencing. Mol. Genet. Metab. Rep..

[211] Palmieri F. (2013). The Mitochondrial Transporter Family SLC25: Identification, Properties and Physiopathology. Mol. Asp. Med..

[212] Ali M.S., Suda K., Kowada R., Ueoka I., Yoshida H., Yamaguchi M. (2020). Neuron-Specific Knockdown of Solute Carrier Protein SLC25A46a Induces Locomotive Defects, an Abnormal Neuron Terminal Morphology, Learning Disability, and Shortened Lifespan. IBRO Rep..

[213] Li Z., Peng Y., Hufnagel R.B., Hu Y.-C., Zhao C., Queme L.F., Khuchua Z., Driver A.M., Dong F., Lu Q.R. (2017). Loss of *SLC25A46* Causes Neurodegeneration by Affecting Mitochondrial Dynamics and Energy Production in Mice. Hum. Mol. Genet..

[214] Wan J., Steffen J., Yourshaw M., Mamsa H., Andersen E., Rudnik-Schöneborn S., Pope K., Howell K.B., McLean C.A., Kornberg A.J. (2016). Loss of Function of *SLC25A46* Causes Lethal Congenital Pontocerebellar Hypoplasia. Brain.

[215] Zou W., Chen Q., Slone J., Yang L., Lou X., Diao J., Huang T. (2021). Nanoscopic Quantification of Sub-Mitochondrial Morphology, Mitophagy and Mitochondrial Dynamics in Living Cells Derived from Patients with Mitochondrial Diseases. J. Nanobiotechnol..

[216] Obinata H., Watanabe T., Takahashi H., Shimo S., Oda T., Sugimoto A., Niwa S. (2025). SLC-25A46 Regulates Mitochondrial Fusion through the Mitofusin Protein FZO-1 and Is Essential for Maintaining Neuronal Morphology. J. Cell Sci..

[217] Hammer M.B., Ding J., Mochel F., Eleuch-Fayache G., Charles P., Coutelier M., Gibbs J.R., Arepalli S.K., Chong S.B., Hernandez D.G. (2017). SLC25A46 Mutations Associated with Autosomal Recessive Cerebellar Ataxia in North African Families. Neurodegener. Dis..

[218] Vignone D., Gonzalez Paz O., Fini I., Cellucci A., Auciello G., Battista M.R., Gloaguen I., Fortuni S., Cariulo C., Khetarpal V. (2022). Modelling the Human Blood-Brain Barrier in Huntington Disease. Int. J. Mol. Sci..

[219] Silva A.C., Almeida S., Laço M., Duarte A.I., Domingues J., Oliveira C.R., Januário C., Rego A.C. (2013). Mitochondrial Respiratory Chain Complex Activity and Bioenergetic Alterations in Human Platelets Derived from Pre-Symptomatic and Symptomatic Huntington’s Disease Carriers. Mitochondrion.

[220] Kumari S., Mehta S.L., Li P.A. (2012). Glutamate Induces Mitochondrial Dynamic Imbalance and Autophagy Activation: Preventive Effects of Selenium. PLoS ONE.

[221] Wang W., Zhang F., Li L., Tang F., Siedlak S.L., Fujioka H., Liu Y., Su B., Pi Y., Wang X. (2015). MFN2 Couples Glutamate Excitotoxicity and Mitochondrial Dysfunction in Motor Neurons. J. Biol. Chem..

[222] López-Molina L., Pereda-Velarde A., di Franco N., Aerts I., Sebastià E., Valls-Roca L., Guitart-Mampel M., Garrabou G., Gines S. (2025). Mitochondria from Huntington’s Disease Striatal Astrocytes Are Hypermetabolic and Compromise Neuronal Branching. Cell Commun. Signal..

[223] Khodorov B. (2004). Glutamate-Induced Deregulation of Calcium Homeostasis and Mitochondrial Dysfunction in Mammalian Central Neurones. Prog. Biophys. Mol. Biol..

[224] Rosenstock T.R., Bertoncini C.R.A., Teles A.V., Hirata H., Fernandes M.J.S., Smaili S.S. (2010). Glutamate-Induced Alterations in Ca^2+^ Signaling Are Modulated by Mitochondrial Ca^2+^ Handling Capacity in Brain Slices of R6/1 Transgenic Mice. Eur. J. Neurosci..

[225] Goubert E., Mircheva Y., Lasorsa F.M., Melon C., Profilo E., Sutera J., Becq H., Palmieri F., Palmieri L., Aniksztejn L. (2017). Inhibition of the Mitochondrial Glutamate Carrier SLC25A22 in Astrocytes Leads to Intracellular Glutamate Accumulation. Front. Cell. Neurosci..

[226] Yang L., Slone J., Li Z., Lou X., Hu Y.-C., Queme L.F., Jankowski M.P., Huang T. (2020). Systemic Administration of AAV-*Slc25a46* Mitigates Mitochondrial Neuropathy in *Slc25a46-/-* Mice. Hum. Mol. Genet..

[227] Sarzi E., Seveno M., Piro-Mégy C., Elzière L., Quilès M., Péquignot M., Müller A., Hamel C.P., Lenaers G., Delettre C. (2018). *OPA1* Gene Therapy Prevents Retinal Ganglion Cell Loss in a Dominant Optic Atrophy Mouse Model. Sci. Rep..

[228] Wang Y., Wu L.-H., Hou F., Wang Z.-J., Wu M.-N., Hölscher C., Cai H.-Y. (2024). Mitochondrial Calcium Uniporter Knockdown in Hippocampal Neurons Alleviates Anxious and Depressive Behavior in the 3XTG Alzheimer’s Disease Mouse Model. Brain Res..

[229] Johnson G.A., Krishnamoorthy R.R., Stankowska D.L. (2023). Modulating Mitochondrial Calcium Channels (TRPM2/MCU/NCX) as a Therapeutic Strategy for Neurodegenerative Disorders. Front. Neurosci..

[230] Fang E.F., Hou Y., Palikaras K., Adriaanse B.A., Kerr J.S., Yang B., Lautrup S., Hasan-Olive M.M., Caponio D., Dan X. (2019). Mitophagy Inhibits Amyloid-β and Tau Pathology and Reverses Cognitive Deficits in Models of Alzheimer’s Disease. Nat. Neurosci..

[231] Fan R., Kim N.-G., Gumbiner B.M. (2013). Regulation of Hippo Pathway by Mitogenic Growth Factors via Phosphoinositide 3-Kinase and Phosphoinositide-Dependent Kinase-1. Proc. Natl. Acad. Sci. USA.

[232] Castellazzi M., Patergnani S., Donadio M., Giorgi C., Bonora M., Bosi C., Brombo G., Pugliatti M., Seripa D., Zuliani G. (2019). Autophagy and Mitophagy Biomarkers Are Reduced in Sera of Patients with Alzheimer’s Disease and Mild Cognitive Impairment. Sci. Rep..

[233] Hou Y., Wei Y., Lautrup S., Yang B., Wang Y., Cordonnier S., Mattson M.P., Croteau D.L., Bohr V.A. (2021). NAD+ Supplementation Reduces Neuroinflammation and Cell Senescence in a Transgenic Mouse Model of Alzheimer’s Disease via cGAS-STING. Proc. Natl. Acad. Sci. USA.

[234] Mills K.F., Yoshida S., Stein L.R., Grozio A., Kubota S., Sasaki Y., Redpath P., Migaud M.E., Apte R.S., Uchida K. (2016). Long-Term Administration of Nicotinamide Mononucleotide Mitigates Age-Associated Physiological Decline in Mice. Cell Metab..

[235] Brakedal B., Dölle C., Riemer F., Ma Y., Nido G.S., Skeie G.O., Craven A.R., Schwarzlmüller T., Brekke N., Diab J. (2022). The NADPARK Study: A Randomized Phase I Trial of Nicotinamide Riboside Supplementation in Parkinson’s Disease. Cell Metab..

[236] Porta F., Siri B., Chiesa N., Ricci F., Nika L., Sciortino P., Spada M. (2021). SLC25A19 Deficiency and Bilateral Striatal Necrosis with Polyneuropathy: A New Case and Review of the Literature. J. Pediatr. Endocrinol. Metab..

[237] Lindhurst M.J., Fiermonte G., Song S., Struys E., De Leonardis F., Schwartzberg P.L., Chen A., Castegna A., Verhoeven N., Mathews C.K. (2006). Knockout of Slc25a19 Causes Mitochondrial Thiamine Pyrophosphate Depletion, Embryonic Lethality, CNS Malformations, and Anemia. Proc. Natl. Acad. Sci. USA.

[238] Jiang Z. (2024). SLC25A19 Is Required for NADH Homeostasis and Mitochondrial Respiration. Free Radic. Biol. Med..

[239] Samur B.M., Gümüş G., Canpolat M., Gümüş H., Per H., Cağlayan A.O. (2022). Clinical and Genetic Studies of Thiamine Metabolism Dysfunction Syndrome-4: Case Series and Review of the Literature. Clin. Dysmorphol..

[240] Ha J., Choi D.-W., Kim K.J., Kim K.Y., Nam C.M., Kim E. (2023). Pioglitazone Use and Reduced Risk of Dementia in Patients with Diabetes Mellitus with a History of Ischemic Stroke. Neurology.

[241] Chandra A., Sharma A., Calingasan N.Y., White J.M., Shurubor Y., Yang X.W., Beal M.F., Johri A. (2016). Enhanced Mitochondrial Biogenesis Ameliorates Disease Phenotype in a Full-Length Mouse Model of Huntington’s Disease. Hum. Mol. Genet..

[242] Baek S.H., Park S.J., Jeong J.I., Kim S.H., Han J., Kyung J.W., Baik S.-H., Choi Y., Choi B.Y., Park J.S. (2017). Inhibition of Drp1 Ameliorates Synaptic Depression, Aβ Deposition, and Cognitive Impairment in an Alzheimer’s Disease Model. J. Neurosci..

[243] Weigele J., Zhang L., Franco A., Cartier E., Dorn G.W. (2024). Sensory-Motor Neuropathy in Mfn2 T105M Knock-in Mice and Its Reversal by a Novel Piperine-Derived Mitofusin Activator. J. Pharmacol. Exp. Ther..

[244] Ma H., Liu Y., Tang L., Ding H., Bao X., Song F., Zhu M., Li W. (2019). Echinacoside Selectively Rescues Complex I Inhibition-Induced Mitochondrial Respiratory Impairment via Enhancing Complex II Activity. Neurochem. Int..

[245] Ou Z., You Y., Yi H., Liu X., Tong Y., Liu D., Wang J. (2024). Key Lipoprotein Receptor Targeted Echinacoside-Liposomes Effective Against Parkinson’s Disease in Mice Model. Int. J. Nanomed..

[246] Wang G., Lu W., Shen W.-B., Karbowski M., Kaushal S., Yang P. (2024). Small Molecule Activators of Mitochondrial Fusion Prevent Congenital Heart Defects Induced by Maternal Diabetes. JACC Basic. Transl. Sci..

[247] Qiu J., Chen Y., Zhuo J., Zhang L., Liu J., Wang B., Sun D., Yu S., Lou H. (2022). Urolithin A Promotes Mitophagy and Suppresses NLRP3 Inflammasome Activation in Lipopolysaccharide-Induced BV2 Microglial Cells and MPTP-Induced Parkinson’s Disease Model. Neuropharmacology.

[248] Gautam M., Genç B., Helmold B., Ahrens A., Kuka J., Makrecka-Kuka M., Günay A., Koçak N., Aguilar-Wickings I.R., Keefe D. (2023). SBT-272 Improves TDP-43 Pathology in ALS Upper Motor Neurons by Modulating Mitochondrial Integrity, Motility, and Function. Neurobiol. Dis..

[249] Quintana-Cabrera R., Quirin C., Glytsou C., Corrado M., Urbani A., Pellattiero A., Calvo E., Vázquez J., Enríquez J.A., Gerle C. (2018). The Cristae Modulator Optic Atrophy 1 Requires Mitochondrial ATP Synthase Oligomers to Safeguard Mitochondrial Function. Nat. Commun..

[250] Noguchi M., Kohno S., Pellattiero A., Machida Y., Shibata K., Shintani N., Kohno T., Gotoh N., Takahashi C., Hirao A. (2023). Inhibition of the Mitochondria-Shaping Protein Opa1 Restores Sensitivity to Gefitinib in a Lung Adenocarcinomaresistant Cell Line. Cell Death Dis..

[251] Djalalvandi A., Takeda K., Grespi F., Fan H., Fonseca T.B., Nogara L., Sharifi S., Barison C., Semenzato M., Omori A. (2025). Opantimirs: A Class of Antagonizing microRNAs That Upregulate Opa1 and Improve Mitochondrial and Disuse Myopathies. Cell Rep. Med..

[252] La Vecchia S., Doshi S., Antonoglou P., Kundu T., Al Santli W., Avrampou K., Witkowski M.T., Pellattiero A., Magrin F., Ames K. (2025). Small-Molecule OPA1 Inhibitors Reverse Mitochondrial Adaptations to Overcome Therapy Resistance in Acute Myeloid Leukemia. Sci. Adv..

[253] Pellattiero A., Quirin C., Magrin F., Sturlese M., Fracasso A., Biris N., Herkenne S., Cendron L., Gavathiotis E., Moro S. (2025). Small Molecule OPA1 Inhibitors Amplify Cytochrome c Release and Reverse Cancer Cells Resistance to Bcl-2 Inhibitors. Sci. Adv..

[254] Babenko V.A., Silachev D.N., Popkov V.A., Zorova L.D., Pevzner I.B., Plotnikov E.Y., Sukhikh G.T., Zorov D.B. (2018). Miro1 Enhances Mitochondria Transfer from Multipotent Mesenchymal Stem Cells (MMSC) to Neural Cells and Improves the Efficacy of Cell Recovery. Molecules.

[255] Cheng X.-Y., Biswas S., Li J., Mao C.-J., Chechneva O., Chen J., Li K., Li J., Zhang J.-R., Liu C.-F. (2020). Human iPSCs Derived Astrocytes Rescue Rotenone-Induced Mitochondrial Dysfunction and Dopaminergic Neurodegeneration in Vitro by Donating Functional Mitochondria. Transl. Neurodegener..

[256] Falzoni S., Vultaggio-Poma V., Chiozzi P., Tarantini M., Adinolfi E., Boldrini P., Giuliani A.L., Morciano G., Tang Y., Gorecki D.C. (2024). The P2X7 Receptor Is a Master Regulator of Microparticle and Mitochondria Exchange in Mouse Microglia. Function.

[257] Kubat G.B., Picone P., Tuncay E., Aryan L., Girgenti A., Palumbo L., Turkel I., Akat F., Singh K.K., Nuzzo D. (2025). Biotechnological Approaches and Therapeutic Potential of Mitochondria Transfer and Transplantation. Nat. Commun..

[258] Morciano G., Pellielo G., Agyapong E.D., Pellegrino C., Patergnani S., Franceschini D., Koutsikos K., Rigon L., Pinton P., Rimessi A. (2026). The Importance of Mitochondria and Mitochondrial Calcium Signaling in Health and Disease: An Updated Outlook on Inflammation. J. Transl. Med..

[259] Sweetat S., Nitzan K., Suissa N., Haimovich Y., Lichtenstein M., Zabit S., Benhamron S., Akarieh K., Mishra K., Barasch D. (2023). The Beneficial Effect of Mitochondrial Transfer Therapy in 5XFAD Mice via Liver-Serum-Brain Response. Cells.

[260] Peruzzotti-Jametti L., Bernstock J.D., Willis C.M., Manferrari G., Rogall R., Fernandez-Vizarra E., Williamson J.C., Braga A., van den Bosch A., Leonardi T. (2021). Neural Stem Cells Traffic Functional Mitochondria via Extracellular Vesicles. PLoS Biol..

